# Thiourea priming enhances salt tolerance through co-ordinated regulation of microRNAs and hormones in *Brassica juncea*

**DOI:** 10.1038/srep45490

**Published:** 2017-04-06

**Authors:** Ashish Kumar Srivastava, Gaurav Sablok, Michael Hackenberg, Uday Deshpande, Penna Suprasanna

**Affiliations:** 1 Nuclear Agriculture and Biotechnology Division, Bhabha Atomic Research Centre, Mumbai 400085, India; 2 Climate Change Cluster (C3), University of Technology Sydney, PO Box 123, Broadway, NSW 2007, Australia; 3 Department of Biodiversity and Molecular Ecology, Research and Innovation Centre, Fondazione Edmund Mach, Via E. Mach 1, 38010 San Michele all’Adige, Trento, Italy; 4 Department of Genetics, Faculty of Sciences, University of Granada, Granada, 1s8071, Spain; 5 Cancer Genetics India (Bioserve), CNR complex, Mallapur Road, Hyderabad - 500076, India

## Abstract

Activation of stress tolerance mechanisms demands transcriptional reprogramming. Salt stress, a major threat to plant growth, enhances ROS production and affects transcription through modulation of miRNAs and hormones. The present study delineates salt stress ameliorating action of thiourea (TU, a ROS scavenger) in *Brassica juncea* and provides mechanistic link between redox, microRNA and hormones. The ameliorative potential of TU towards NaCl stress was related with its ability to decrease ROS accumulation in roots and increase Na^+^ accumulation in shoots. Small RNA sequencing revealed enrichment of down-regulated miRNAs in NaCl + TU treated roots, indicating transcriptional activation. Ranking analysis identified three key genes including *BRX4, CBL10* and *PHO1*, showing inverse relationship with corresponding miRNA expression, which were responsible for TU mediated stress mitigation. Additionally, ABA level was consistently higher till 24 h in NaCl, while NaCl + TU treated roots showed only transient increase at 4 h suggesting an effective stress management. Jasmonate and auxin levels were also increased, which prioritized defence and facilitated root growth, respectively. Thus, the study highlights redox as one of the “core” components regulating miRNA and hormone levels, and also strengthens the use of TU as a redox priming agent for imparting crop resilience to salt stress.

Plants perceive and respond to a variety of environmental stresses using a plethora of signalling cascades which allows them to survive under challenging environmental conditions. Among widely reported ecological abiotic stresses, salt stress is considered as one of the important factors responsible for decreasing crop productivity worldwide. Thus, it is imperative to understand molecular mechanism underlying salt tolerance so as to minimize the negative impacts, which could lead to 50% arable land loss by 2050 due to increased salinization^1^,^2^,^3^. Previous reports have identified several molecular regulators of salt tolerance having the potential to develop salt-tolerant genotypes^4^,^5^,^6^,^7^,^8^; however, their implementation for crop improvement in realistic field conditions is still a long-term goal. To complement genetic approach, chemical priming has been postulated as an alternative strategy which can enhance plant’s ability to tolerate different types of abiotic stresses^9^,^10^,^11^,^12^. Although, most of the priming agents have their own chemical-specific effects, a unified mechanism for their mode of action has been recently proposed which is based upon their ability to modulate redox state homeostasis^13^.

Cellular redox state is described as an integrated ratio of reduced to oxidized form of all the redox couples present inside the cell and has been shown to be regulated by reactive oxygen species (ROS) scavenging/producing enzymes and antioxidant metabolites^14^. Recently, redox homeostasis is proposed as a central regulator of stress signalling, including those under salt stress^15^,^16^. Additionally, the oxidation-reduction based redox reactions are considered as intrinsic part of plant metabolism^17^,^18^,^19^ and hence, stress induced redox imbalance negatively affects multiple plant processes including growth, development and stress tolerance. In view of this, we tested the ability of thiourea (TU; a non-physiological thiol based ROS scavenger^20^,^21^) to restrict stress induced redox imbalance and associated damages in plants. The ameliorative potential was demonstrated at different organizational levels in lab as well as field conditions. At physiological level, TU improved source-to-sink relationship leading to increased crop yield^22^,^23^ and at molecular level, it improved cellular energetics^24^, co-ordinated calcium and abscisic acid (ABA) signaling events^25^, maintained plant-water homeostasis^26^, enhanced antioxidant defense^27^ and improved sulphur metabolism^28^. The involvement of several diverse pathways in TU mediated stress mitigation further indicated the significance of redox homeostasis for regulating multiple processes in plants.

The activation of stress response mechanisms demands an extensive transcriptional reprogramming. There is a class of small non-coding RNAs called microRNAs, which regulates gene expression, at post-transcriptional level, either through transcript cleavage or translational inhibition^29^. Although, the role of miRNAs as salt-regulators is well-perceived^30^, the set of miRNAs regulated through cellular redox state has not been described. Apart from miRNAs, different types of plant hormones such as auxin, ABA and jasmonic acid are also considered as important regulators of salt tolerance in plants^31^,^32^,^33^,^34^. The hormonal and redox signaling crosstalk has been demonstrated under different abiotic stress conditions^35^; however, little is known in *Brassica juncea*, especially under the context of salt stress.

*Brassica juncea* belongs to the family Brassicaeace and represent classical example of recursive whole genome duplication events. It is an allotetraploid (AABB) oilseed crop from the triangle of U (*B. rapa* (AA) and *B. nigra* (BB) and allotetraploids *B. napus* (AACC) and *B. carinata* (BBCC)^36^; of which, *B. rapa* genome is currently available. *B. juncea* contributes to 12% of total world edible oil production (http://faostat.fao.org/). Salt stress has been described as a major factor which negatively influences the growth and oil production in *Brassica* species^37^,^38^,^39^. The present study highlights the mechanistic basis of TU mediated salt tolerance at the level of miRNA and hormone based regulations. The research outcome not only advances our understanding about the concept of redox mediated regulation of salt-tolerance; but, also strengthens the applicability/adoptability of TU based technology for enhancing crop protection.

## Results and Discussion

Increasing salinization and decrease in the arable land necessitates the mechanistic understanding of salt tolerance so that suitable crop improvement strategies can be developed^1^,^2^,^3^. Towards this endeavour, TU supplementation has been widely used to minimize negative effects of salt stress in different crop plants^13^. TU is a potent redox scavenger and has ability to scavenge multiple ROS including superoxide radical and hydrogen peroxide (H_2_O_2_)^20^,^21^. Realizing its ROS scavenging capacity, TU supplementation has also been utilized, as a chemical probe, to understand redox regulatory components associated with salt^24^,^25^,^26^,^27^ and arsenic stress^28^ tolerance in plants. However, not much information is known about post-transcriptional and hormone based regulations associated with TU mediated response. The present study was performed to understand how TU modulates miRNAs and different hormones to activate tolerance mechanisms under salt-stress conditions. Owing to ROS/redox mediated action of TU, the identified miRNAs and mode of hormone regulation could be classified as “redox-regulated” in the context of salt stress. Although, the same objective can also be achieved using other redox-active molecules such as ascorbate, glutathione and cysteine; however, TU, being a non-physiological thiol, the effects produced are more closely associated with cellular redox state^28^.

### TU improves plant growth phenotype under salt stress

**Figure 1:**
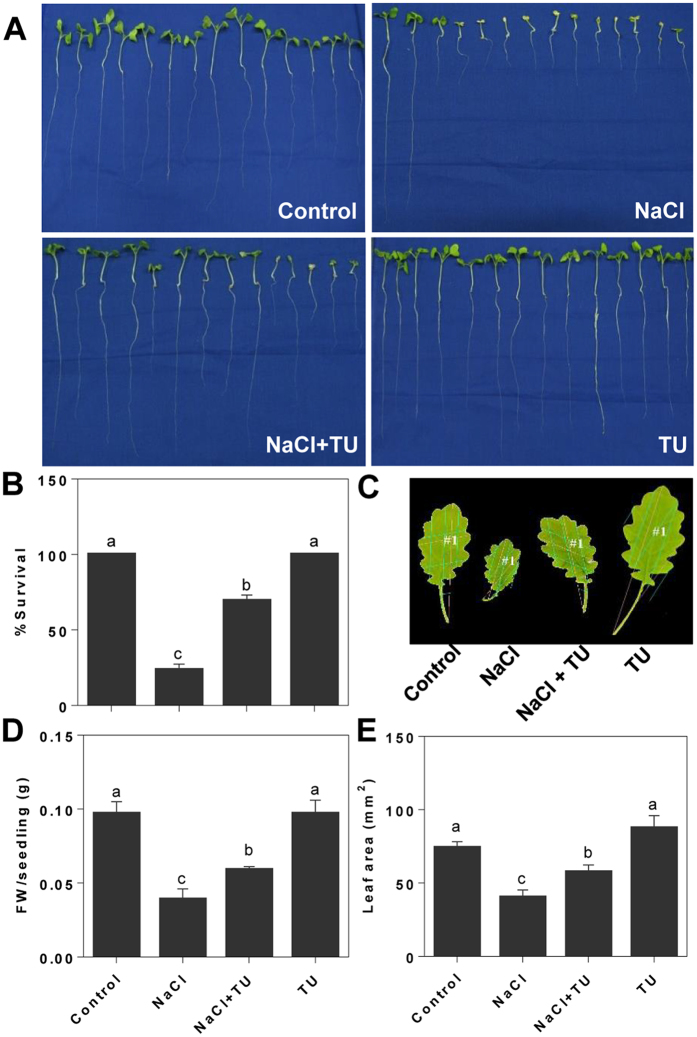
Differential phenotype of *B. juncea* seedlings with/without thiourea (TU) treatment. The seedlings were grown hydroponically under control condition for 10 d and then subjected to different treatments such as control (1/2 MS), NaCl (150 mM), NaCl (150 mM) + TU (75 μM) and TU (75 μM). For NaCl + TU and TU alone treatments, 24 h pre-treatment with same concentration of TU was also given. At 7 d after treatment, differential phenotype was recorded qualitatively (**A**) and quantified in terms of survival efficiency (**B**). In order to obtain leaf phenotype, NaCl concentration was reduced to 125 mM and phenotype was recorded at 10 d after treatment both qualitatively (**C**) and quantitatively in terms of fresh weight per seedling (**D**) and leaf area (**E**). The data represents the mean ± SE of three biological replicates. Different letters on bar graph have been put on the basis of LSD value derived from SPSS software (DMRT, P < 0.05).

To assess the phenotypic differences, in terms of survival and growth, post-germination phenotyping was performed on hydroponically grown seedlings under NaCl with/without TU treatment. We observed significant increase in survival efficiency in NaCl + TU (69%) as compared with NaCl (24%) treatment at 150 mM NaCl concentration (Fig. 1A,B). The growth phenotype was evaluated at 125 mM NaCl concentration. A greater decrease in biomass accumulation was observed in NaCl (60%) than NaCl + TU (39%) treatment, as compared with that of control (Fig. 1D). This was simultaneous with significant increase in average leaf area by 42% in NaCl + TU as compared with NaCl treatment (Fig. 1C and E). Thus, the observed phenotype clearly demonstrates the ameliorative potential of TU against NaCl stress induced toxicity.

### TU lowers ROS accumulation and improves ionic balance in plants

**Figure 2:**
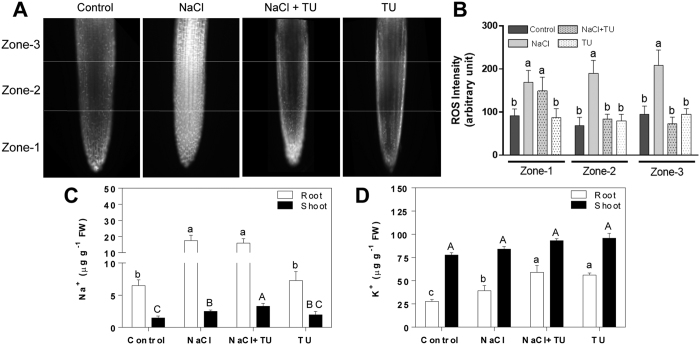
Differential accumulation of ROS and quantification of Na^+^ and K^+^ ions in *B. juncea* seedlings. The 15 d old hydroponically grown seedlings were given different treatments such as control (1/2 MS), NaCl (125 mM), NaCl (125 mM) + TU (75 μM) and TU (75 μM). For NaCl + TU and TU alone treatments, 24 h pre-treatment with same concentration of TU was also given. The ROS accumulation in roots was checked at 4 h after treatment using DCF-DA staining (**A**). The root region was divided into three different zones and ROS intensity was quantified independently using Image J program (**B**). A total of 15 seedlings were stained for each treatment and experiment was repeated twice to check its reproducibility. The cations like Na^+^ (**C**) and K^+^ (**D**) were measured at 24 h after treatment in both root and shoot. The data represents the mean ± SE of three biological replicates. Different letters on bar graph have been put on the basis of LSD value derived from SPSS software (DMRT, P < 0.05). Small and capital case letter is used for root and shoot, respectively.

Abiotic stresses including NaCl are known to increase ROS production^7^. In *Arabidopsis thaliana*, it has been shown that 3 h treatment duration was sufficient to produce maximal changes in redox and beyond that, even incubating upto 3 days in salt does not shift redox more towards oxidizing conditions^40^. Besides, 3–4 h time duration has been widely used to delineate stress induced signalling network in plants^41^,^42^. Keeping this into account, we measured ROS accumulation at 4 h time point in root-tip region under different treatment conditions. In order to have high-resolution, measurement region was further divided into three different zones with zone-1 starting from root-tip (Fig. 2A). A sharp increase in ROS accumulation was observed in zone-1 under both NaCl and NaCl + TU treatments. However, in zone-2 and 3, ROS level was decreased by 2.2- and 2.9-fold, respectively in NaCl + TU as compared with NaCl treatment (Fig. 2B). No significant difference in ROS level was observed in TU alone treatment as compared to that in control. The data confirmed *in planta* ROS scavenging ability of TU which has already been demonstrated in shoot^27^ and along root-axis^26^.

In order to correlate TU mediated ameliorative phenotype with the activation of salt tolerance mechanism; we measured Na^+^ and K^+^ ion status in root and shoot under different treatments. In roots, Na^+^ levels increased equally in NaCl and NaCl + TU treatments; however, in shoot, it was increased more in NaCl + TU (2.2-fold) than NaCl (1.7-fold) treatment, as compared with that of control (Fig. 2C). This suggested the possibility that TU supplementation could accelerate Na^+^ translocation towards shoots which is considered as one of the important defence mechanism to avoid *built up* of Na^+^ toxicity in roots^43^. Recently, comparative evaluation of contrasting rice varieties have confirmed that tolerant varieties have better potential of translocating Na^+^ from root-to-shoot^44^. Although, no significant change in K^+^ was observed in shoot; in roots, it was maintained at significantly higher level of 1.42−, 2.13− and 2-fold in NaCl, NaCl + TU and TU treatments, respectively, as compared with control (Fig. 2D). The increased K^+^ level in NaCl treated roots indicated that osmotic effects were pre-dominant at 24 h after stress exposure which caused membrane hyperpolarization leading to increased K^+^ uptake^45^. The further increase in K^+^ level upon TU supplementation could be related to reduced K^+^ efflux under reducing conditions^46^. Such a change in the levels of cations, suggest that apart from ameliorating Na^+^ toxicity, TU supplementation might also tackle K^+^ deficiency; however, this needs to be validated further.

### TU mediated effect is regulated post-transcriptionally through miRNAs

To link observed phenotypic changes with miRNA mediated post-transcriptional regulation, size selected smallRNAs libraries ranging from 18 to 30 nt were sequenced from seedlings under different treatments, independently in root and shoot. The observed size distributions of adapter cleaned reads showed classes from 21–23 nt with majority of 23 nt, followed by 21nt, and 22nt (Supplementary Figure 1) which was similar to previously reported size distribution in plants^47^,^48^. The conserved miRNAs were identified by mapping cleaned reads to miRBase. However, due to the non-availability of reference genome, and taking into account of close conservation between *B. rapa* and *B. juncea*, we used a cross-species mapping approach to identify novel miRNAs. Interestingly, a high proportionate of unique read mapping was observed both in root and shoot (Supplementary Table 1), which can be further related to evolutionary conservation across *Brassica* triangle of U^49^. Overview heat-map of conserved and novel miRNAs expression is shown in Supplementary Figure 2.

To understand miRNA mediated regulation in different treatments, we followed the following approach. Initially, conserved miRNAs showing differential expression in at least one treatment based on statistical significance (p < 0.05) were identified. Further, those miRNAs showing differential expression based upon their expression significance (logFC ≥ 1) was also mined. Cumulative lists of differentially expressed conserved miRNAs for root (Supplementary Table 2) and shoot (Supplementary Table 3) are shown. Although, a set of 18 novel high confidence (HC) miRNAs were identified; however, due to cross-mapping approach, low read count abundance was obtained which lowered the number of differentially expressed novel miRNAs after putting stringent p-value and fold-change cutoffs. All the predicted novel miRNAs were amplified using stem-loop PCR and amplicons were sequenced. Out of 18, we could confirm the mature sequence for 5 novel miRNAs (termed as bra-mirX1–5; refer Supplementary Table-4 for mature and hairpin sequence). The specific amplification was also validated through melting curve analysis (Supplementary Figure 3). Later on, the expression levels for these five high-confidence sequence validated novel miRNAs were measured using stem-loop PCR (Supplementary Table 5).

**Figure 3:**
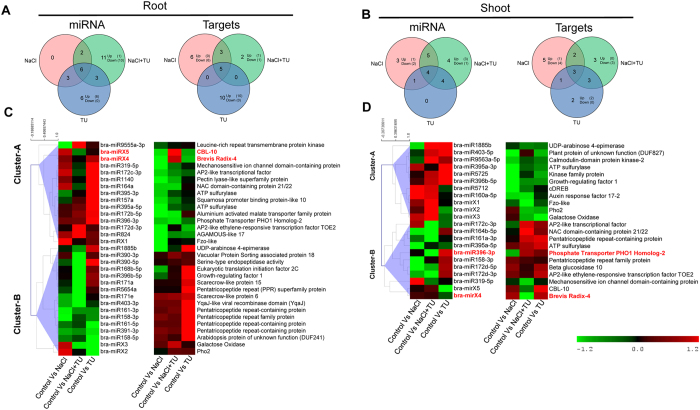
Distribution and differential expression of conserved/novel miRNAs and corresponding targets in *B. juncea* seedlings. The seedlings were grown hydroponically for 15 d and then subjected to different treatments such as control (1/2 MS), NaCl (125 mM), NaCl (125 mM) + TU (75 μM) and TU (75 μM). For NaCl + TU and TU alone treatments, 24 h pre-treatment with same concentration of TU was also given. At 4 h after treatment, root and shoot were harvested independently and subjected to small RNA sequencing using SOLiD platform. The differentially expressed miRNAs and target genes under different treatments were analyzed using venn-diagram, independently for root (**A**) and shoot (**B**). The heat-map represents the average change in miRNA and corresponding target gene expression from two biological replicates. The data was clustered into two distinct sets on the basis of up- (Cluster-A) and down-regulated (Cluster-B) miRNAs. The ranking analysis was performed using expression difference of target genes between NaCl + TU and TU and top-ranked genes along with corresponding miRNAs are highlighted as **“bold red”**. The absolute expression change can be found as Supplementary Table 10 (root) and 11 (shoot).

The expression data of conserved (on the basis of RNAseq) and novel miRNAs (on the basis of stem-loop) were pooled together and their expression pattern was analysed using venn-diagram, independently for root (Fig. 3A and Supplementary Table 6) and shoot (Fig. 3B and Supplementary Table 7). The miRNA pattern in different treatments was found distinct as early as 4 h after treatment, which suggested that miRNA expression is very versatile and can be seen as the direct reflection of external treatment condition. For the entire set of differentially expressed miRNAs, target gene expressions were quantified through quantitative real-time PCR and analyzed using venn-diagram, independently for root (Fig. 3A and Supplementary Table 8) and shoot (Fig. 3B and Supplementary Table 9). In both the organs, proportion of elements shared between NaCl and NaCl + TU treatment were found more than NaCl + TU specific elements which clearly indicated that TU effect on restoration of gene expression was only partial. This also explained why we did not observe complete amelioration of growth phenotype under NaCl + TU treatment (Fig. 1). The expression data of miRNAs along with corresponding target genes were clustered together which yielded two distinct clusters: cluster-A and B representing up- and down-regulated miRNAs, respectively. The overview heat-map for these clusters reveals an inverse relationship between miRNA and target gene expression in root (Fig. 3C) and shoot (Fig. 3D). This validated the accuracy and robustness of target gene prediction pipeline used in the present study.

A large number of miRNAs were found to be upregulated specifically in TU alone treated roots (Fig. 3A). This suggested an overall decrease in protein turnover rate under reducing redox environment. The higher miRNA expression will lower down their mRNA to avoid unnecessary protein synthesis. In contrast, no TU specific miRNA was found to be present in shoot (Fig. 3B).

### TU dependent activation of defence genes during early stage NaCl exposure in roots

The miRNA expression pattern in roots revealed 10 miRNAs which showed significant down-regulation specifically under NaCl + TU treatment (Fig. 3A). This included eight conserved (bra-miR171e, bra-miR390–3p, bra-miR403-3p, bra-miR168b-5p, bra-miR171a, bra-miR390-5p, bra-miR396b-5p and bra-miR5654a) and two novel (bra-miRX1 and bra-miRX5) miRNAs (Supplementary Table 6). Out of 10, for four miRNAs such as bra-miR168b-5p, bra-miR396b-5p, bra-miRX1 and bra-miRX5, corresponding target genes such as eukaryotic translation initiation factor 2 C (*eIF2C*), growth regulating factor 1 (*GRF1*), FZO-like (*FZO*) and calceneurin B-like 10 (*CBL10*), respectively, showed upregulated expression in NaCl + TU, as compared with NaCl treatment. For remaining six miRNAs, no significant difference in target gene expression was observed between NaCl and NaCl + TU treatment (Supplementary Table 10). This suggested that apart from miRNA abundance, target gene expression can also be regulated through other factors such as availability of co-factors and overall cellular redox environment. The up-regulated targets have been demonstrated to mediate salt tolerance in plants. For instance, *eIF2c* (targeted through bra-miR168b-5p) is responsible for maintaining protein synthesis rate and overall plant growth. Few translation factors such as eIF1A^50^ and eIF5A^51^ are known to impart abiotic stress tolerance. *GRF1* (targeted through bra-miR396b-5p) belongs to the small family of transcription factor which modulates growth through its interaction with gibberellins^52^. *FZO* (targeted through bra-miRX1) codes for a dynamin-related membrane-remodelling protein targeted to chloroplasts and specifically to thylakoid membranes and previously *fzo* mutants have been shown to have disorganized chloroplast^53^. FZO function has been demonstrated to restore photosynthetic electron system under salt stress^54^. *CBL10* (targeted through bra-miRX7) codes for a calcium sensor which interacts with CIPK27 and mediates Na^+^ ion sequestration inside the vacuoles^55^. Among other targets with upregulated expression in NaCl + TU included brevis radix-4 (*BRX4*; targeted through bra-miRX4), ATP sulphurylase (*APS*; targeted through bra-miR395-3p), phosphate-2 transporter (*PHO2*; targeted through bra-miRX2), galactose oxidase (*GO*; targeted through bra-miRX3) and UDP-arabinose 4-epimerase (*UDPase*; targeted through bra-miR1885b). BRX is a key regulator of root meristem activity and *brx* mutant in Arabidopsis are impaired in primary root growth^56^. APS is an important enzyme which catalyses the committed step of sulphur assimilation^57^. Since, the involvement of sulphur metabolism in TU mediated amelioration against arsenic stress has already been demonstrated^28^; APS upregulation under NaCl + TU and TU treatments are justified. The upregulated expression of targets such as *PHO2, GO* and *UDPase* suggested the modulation of nucleotide sugar metabolism which might support the growth of seedlings under NaCl + TU treatment. Thus, TU dependent ameliorative growth phenotype under NaCl stress is associated with upregulated expression of wide-range of defence genes.

### Activation of contrasting responses in NaCl and NaCl + TU treated shoots

Like roots, miRNA expression pattern in shoot was also found distinct under different treatment conditions; however, unlike roots, less number of differentially induced miRNAs was present in shoot (Fig. 3B). This could be due to the fact that miRNAs profiling was done at 4 h after treatment and roots, being the first organ to come in direct contact with stress, have shown more responsiveness than shoots. Furthermore, these organs have high level of functional specialization and hence, their internal redox environment, co-factor and metabolite compositions, which are known to affect miRNA expression, are expected to be different.

The targets showing upregulated expression in NaCl + TU were related to either DNA or RNA binding functions (Supplementary Table 11). Few of the examples include PPR (pentatricopeptide repeat containing gene; targeted through bra-miR161a-3p) which is a RNA binding protein and facilitate RNA editing or splicing of organelle genes^58^,^59^. *AP2* like transcription factor (targeted through bra-miR172c-3p) codes for a DNA binding protein and regulate G1-S transitions in shoot apical meristem^60^. NAC transcription factor (targeted through bra-miR164b-5p), is a DNA binding protein which regulate stress related gene expression through ABA dependent pathway^61^. On the contrary, targets showing upregulated expression in NaCl treatment were mainly effector genes. For instance, *BRX* (targeted through bra-miRX4) is a rate-limiting gene for regulating auxin-responsive gene expression^62^. Mechanosensitive ion channel protein (targeted through bra-miR319-5p) functions in adapting hyperosmotic shock^63^; while, CBL10 (targeted through bra-miRX7) functions to sequester Na^+^ inside the vacuole. GO (targeted through bra-miRX3) and UDP-arabinose 4-epimerase (targeted through bra-miR1885b) are involved in the regulation of sugar nucleotide signaling. Thus, a clear enrichment of contrasting set of targets was observed under NaCl and NaCl + TU treated shoot. While NaCl + TU activate signaling related targets; NaCl mainly activates defence or adaptive genes. This also suggested that TU could act as booster of signaling under salt stress conditions.

### Deviation from miRNA mediated target gene repression

The miRNA and target gene expressions were also analyzed in terms of Pearson’s correlation coefficient (r) to understand their inter-relationship. We found few targets where r value was positive (in some cases, it was almost close to 1) which suggested that these targets do not follow the universal rule of miRNA mediated repression of gene expression. In roots, such targets showing differential expression between NaCl and NaCl + TU treatment included *APS* and *AP2*-like transcription factor which were targeted through bra-miR395a-5p (r = 0.72) and bra-miR172d-3p (r = 1), respectively. The downregulated expression of *APS* was observed only in NaCl, although, bra-miR395a-5p expression was same in both NaCl and NaCl + TU treatment. *AP2* transcription factor remains upregulated in NaCl + TU treatment, inspite of having an upregulated expression of bra-miR172d-3p (Supplementary Table 10). Similarly, in shoot, bra-miR9563a-5p and its target gene *CDPK2* both showed an upregulated expression in NaCl + TU treatment (r = 1; Supplementary Table 11). Such a deviation from universal mode of miRNA functioning suggest different possibilities. First: it has been recently proposed that apart from miRNA abundance, other cofactors also regulate miRNA mediated target gene cleavage^64^. Since, NaCl and NaCl + TU treatments are known to have differential metabolite accumulation^25^,^27^,^28^; therefore, distinct modes of miRNA regulation in these treatments are expected. Second: low miRNA:mRNA target interaction which is considered as an important mechanism to fine tune gene expression^65^. Additionally, there might also be a time lag between change in miRNA and target gene expression. However, the exact reason behind the decoupling of miRNA:mRNA interaction under NaCl + TU treatment need further investigation. Thus, the results indicated different modes of miRNA mediated regulation through which target gene expressions are regulated under stress conditions.

### Identification of key gene(s) responsible for redox-mediated regulation of salt tolerance

After evaluating the expression profiles of target genes under different treatments, our next objective was to identify key gene(s) which is controlled through miRNA and associated with redox mediated regulation of salt tolerance. To achieve this, initially the expression difference between NaCl + TU and TU was computed and target genes were ranked according to order from highest-to-lowest, independently in root (Supplementary Table 10) and shoot (Supplementary Table 11). Later on, using 2 fold cut-off, we identified top-ranked genes showing maximum difference in expression between NaCl and NaCl + TU treatment. Using this approach, we identified *CBL10* and *BRX4* in root and *PHO1* and *BRX4* in shoot as “key genes” whose differential expression might maximally contribute towards the ameliorative phenotype observed under NaCl + TU treatment. Except *PHO1*, remaining three targets were regulated through novel miRNA identified in the present study. CBL10 is involved in Na^+^ reallocation and regulation of ionic homeostasis in plants^55^. Significantly higher expression of *CBL10* in roots could relate to the increased Na^+^ content observed in NaCl + TU treated shoots as compared with that of NaCl (Fig. 2C). PHO1 is a phosphate exporter and *pho1* mutant show all the symptoms of phosphate deficiency including reduced shoot growth^66^. BRX4 is a member of Brevis-radix family, considered as one of the important gene family for maintaining root growth and overall plant fitness under stress condition^62^,^67^. Upregulated expression of *BRX4* and *CBL10* in root was limited to NaCl + TU while, these genes remain downregulated in TU alone treatment (Supplementary Table 10), suggesting their regulation through both redox and salt stress dependent manner. Since, redox state is one of the “core” regulators of signaling under stress^15^,^16^, the identified targets will have biotechnological relevance for improving salt tolerance in crop plants. Additionally, the expression of these genes was inversely correlated with corresponding miRNA expression in both root (*CBL10*:bra-mirX5, r = −0.91 and *BRX4*:bra-mirX4, r = −0.88) and shoot (*PHO1*: bra-miR396-3p, r = −0.27 and *BRX4*:bra-mirX4, r = −0.45) which suggested that miRNA mediated regulation is one of the major mechanisms to modulate their expression level. Thus, the genetic manipulation of these key genes could also be attempted by changing the level of corresponding miRNAs.

### Improved plant phenotype under NaCl + TU is co-ordinated through hormonal regulations

Hormones are known to play critical role in shaping up plant phenotype under different developmental and stress conditions. Since, hormones are demonstrated to have crosstalk with cellular redox state^35^,^68^; a distinct and differential response of hormones under NaCl and NaCl + TU treatment is expected. Further, out of three key genes identified for redox-mediated regulation of salt tolerance, two of them such as *BRX4* and *PHO1* are known to integrate responses related to multiple plant hormones including auxin^69^ and ABA^66^. Thus, to underpin redox-hormone crosstalk, and to provide biochemical basis for the conclusion drawn from miRNA/target expression profiling, we measured the levels of hormones such as ABA, auxin and jasmonic acid under different treatment conditions.

**Figure 4:**
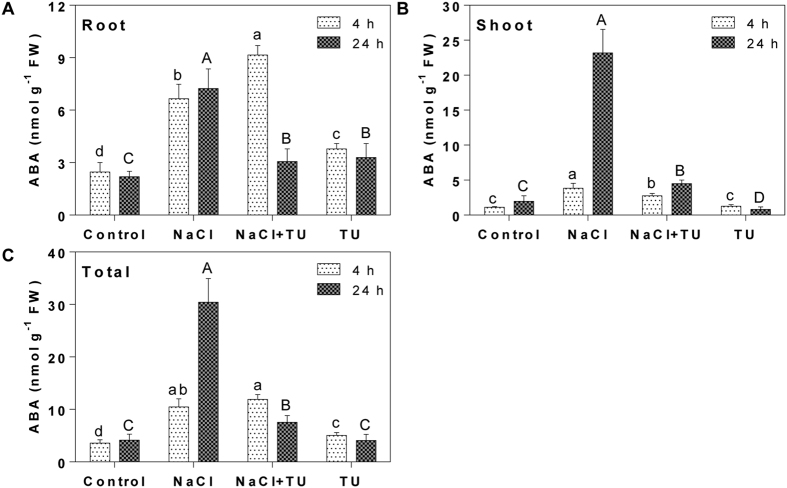
ABA quantification in *B. juncea* seedlings under different treatments. The 15 d old hydroponically grown seedlings were given different treatments such as control (1/2 MS), NaCl (125 mM), NaCl (125 mM) + TU (75 μM) and TU (75 μM) and ABA was quantified at 4 and 24 h after treatment independently in root (**A**) and shoot (**B**). The whole plants ABA represent the sum of root and shoot (**C**). For NaCl + TU and TU alone treatments, 24 h pre-treatment with same concentration of TU was also given. The data represents the mean ± SE of three biological replicates. Different letters on bar graph have been put on the basis of LSD value derived from SPSS software (DMRT, P < 0.05). Small and capital case letter is used for 4 and 24 h, respectively.

At 4 h, ABA level was increased in NaCl and NaCl + TU; however, the observed changes in root and shoot were differential. In NaCl + TU, ABA increased and decreased by 1.37- and 0.72-fold in root and shoot, respectively, as compared with NaCl treatment (Fig. 4A,B). ABA is an important stress hormone and its induction is linked with plant’s ability to mediate salt tolerance^70^. Short-term ABA data support this as whole plant ABA level was increased by 1.13-fold in NaCl + TU as compared with NaCl treatment (Fig. 4C). It also signifies that their relative distribution in root and shoot is important for the activation of salt tolerance. For instance, TU supplementation kept ABA higher in root which can facilitate better NaCl perception and defence activation. This is supported from the fact that salt-tolerant cultivars of maize accumulate higher ABA in root as compared with salt-sensitive cultivar^71^. Lower ABA in NaCl-treated root might be due to its increased root-to-shoot translocation for regulating stomata movement which help to conserve plant water status^72^. Since, TU has been shown positive to regulate plant-water homeostasis^26^, the extent of such translocation is expected to be lower in NaCl + TU and hence, ABA will be increased in root. Upon 24 h NaCl exposure, whole plant ABA in NaCl was 4-fold higher than NaCl + TU (Fig. 4C) with the major accumulation seen in shoot (Fig. 4B). In NaCl and NaCl + TU treated shoot, ABA level was increased by 11.8- and 2.2-fold, respectively as compared with control (Fig. 4B). Significantly lower ABA in NaCl + TU at long-term clearly signifies TU potential towards NaCl stress amelioration. Previously, ABA has been used as an indicator to check ameliorating potential of salt-priming towards air-drying stress^73^. ABA response was also differential in TU alone treatment. In general, ABA was increased in root while in shoot, although no change was detected at 4 h, it was significantly decreased by 0.4-fold at 24 h, as compared with control (Fig. 4). The change in ABA could be attributed to TU mediated modulation of ABA biosynthesis/catabolism related genes demonstrated earlier^25^. Thus, during TU pre-treatment stage, plants maintained higher ABA in roots which help them to respond better during salt stress exposure. Further, in shoot, the processes to lower down ABA were presumably accelerated which avoids the build-up of ABA during long-term stress exposure.

**Figure 5:**
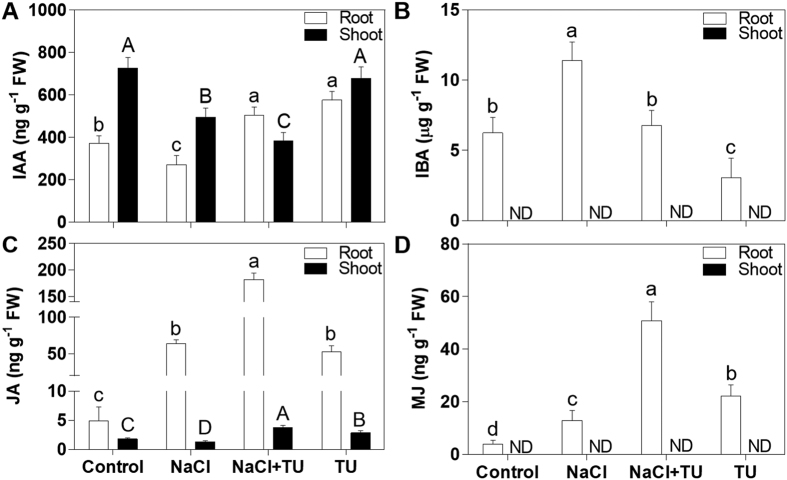
Auxin and jasmonate quantification in *B. juncea* seedlings under different treatments. The 15 d old hydroponically grown seedlings were given different treatments such as control (1/2 MS), NaCl (125 mM), NaCl (125 mM) + TU (75 μM) and TU (75 μM) and at 24 h after treatment, different hormones such as IAA (**A**), IBA (**B**), JA (**C**) and methyl jasmonate (**D**) were quantified in root and shoot. For NaCl + TU and TU alone treatments, 24 h pre-treatment with same concentration of TU was also given. The data represents the mean ± SE of three biological replicates. Different letters on bar graph have been put on the basis of LSD value derived from SPSS software (DMRT, P < 0.05). The representative HLC profile showing the separation and retention time of both the standards are given as Supplementary Figure 4 (for IAA and IBA) and 5 (for JA and MJ). Small and capital case letter is used for root and shoot, respectively. ND stands for not-detectable.

Auxins like IAA (active) and IBA (stored form) have been shown to play important roles in salt tolerance. The NaCl stress mediated increase in ROS level has been demonstrated to reduce IAA which finally reduces the root growth^74^. Similar to this, we also observed 0.7-fold decrease in IAA in NaCl treated roots, as compared with control (Fig. 5A). In contrast, NaCl + TU treated roots maintained 1.35-fold higher IAA level which could explain its better root growth phenotype (Fig. 1). Further, IAA level in shoot was also differential with the higher extent of decrease observed in NaCl + TU as compared with NaCl. This suggested that TU accelerated the directional movement of IAA from shoot-to-root to maintain higher level of IAA in root. Additionally, we also detected IBA in roots and its level was significantly decreased by 0.59-fold in NaCl + TU as compared with NaCl (Fig. 5B). This suggested that IBA-to-IAA conversion was also enhanced to support higher IAA level in roots. Since, this conversion takes place in peroxisomes which is the major site for ROS production inside the cell^75^; therefore, the modulation of IBA-to IAA conversion rate is expected in response to TU treatment. This was supported by 0.48-fold decrease in IBA level with the simultaneous 1.5-fold increase in IAA level in TU alone treatment (Fig. 5A). Thus, TU priming can activate auxin metabolism to ensure higher IAA level in root.

The jasmonates (jasmonic acid, JA-the active form; and methyl jasmonates, MJ-the storage form) are another class of hormones which regulate salt tolerance through crosstalk with other plant hormones^34^. In roots, both MJ and JA were increased under all the treatments; with the maximum JA level being observed in NaCl + TU treated root (Fig. 5C,D). In shoot, JA level was increased by 2- and 1.58-fold in NaCl + TU and TU treatment, respectively; while, in NaCl, it was decreased by 0.74-fold, as compared with control (Fig. 5C). Since, JA has been demonstrated to prioritize defence over growth^76^; the higher accumulation of JA will help in activating defence against salt stress. This is supported by the fact that exogenous JA can activate tolerance in salt-sensitive cell lines of grapes^31^. The specific induction of JA in NaCl + TU could also be explained as antagonistic responses between ABA and JA^77^. Since, both NaCl + TU and TU maintained lower ABA level, therefore, JA induction could takes place; on the contrary, NaCl treatment had higher ABA, which probably did not support JA induction.

Thus, the overall hormone data suggested that TU primed the seedling through redox-homeostasis which help them to temporally regulate their ABA level during short- and long-term stress exposure which might have facilitated the activation of JA mediated defence. Further, auxin level also increased which avoids NaCl induced toxicity on root growth.

### Redox, miRNA and hormone based regulatory module: linear or bifurcative?

After identifying key miRNAs and target genes and evaluating the role of different hormones under the context of salt tolerance, our next objective was to understand the kind of inter-relationship exists between redox regulated miRNAs and hormones. Two different types of model can be proposed in this regard: redox-miRNA-hormone based linear model or redox-miRNA & redox-hormone based bifurcative model. To test these possibilities, we first performed gene set enrichment analysis using miRNA targets, independently for root (Supplementary Table 12) and shoot (Supplementary Table 13). No functional category directly related to hormone biosynthesis or catabolism was found to be enriched which suggested that changes in hormone levels are more or less independent to miRNA or target gene expression. However, DNA or nucleic acid binding functions were found to be enriched under molecular function category which indicated extensive transcriptional reprogramming during early hours of stress which might have an indirect effect on hormone level and *vice versa*. For example, in our case we identified *BRX4* as one of the key targets for redox mediated regulation of salt tolerance. BRX regulates quantitative aspects of root growth in auxin concentration dependent manner^69^. We observed an opposite expression pattern of *BRX4* in root and shoot under different treatments (Supplementary Tables 10 and 11), which exactly coincides with corresponding IAA levels. This was evident with strong positive correlation observed between *BRX4* expression and IAA levels in root (r = 0.51) and shoot (r = 0.80). This suggested the possibility of miRNAs-hormones crosstalk which has been well-demonstrated under multiple plant processes including root development and stress response^78^; however, in most of the cases, miRNAs function has been demonstrated to modulate downstream signaling and not hormone level. On the contrary, redox state has been shown to have more direct relationship with hormone metabolism including that of ABA, JA and auxins^35^. Thus, it strongly appears that redox-miRNA-hormone inter-relationship is not linear; but, both miRNA and hormones are linked through a common point of redox state which acts as core regulator of signaling. Upon redox state fluctuation, parallel processing is initiated which changes miRNA expression as well as hormone level and they together shape the plant phenotype according to the altered condition.

## Conclusion

**Figure 6:**
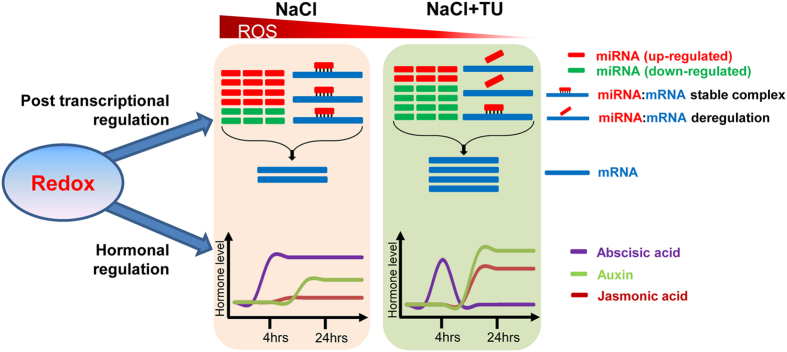
Redox, miRNA and hormone based bifurcative model in *B. juncea*. The redox-state acts as a central point to regulate both miRNA and hormone levels through a bifurcative mechanisms. Owing to its scavenging action, TU supplementation helps in reducing ROS levels in seedling under NaCl treatment. This alters miRNA mediated post-transcriptional regulation by two ways. Firstly, miRNAs expressions were down-regulated and secondly, miRNA:mRNA interaction gets deregulated. As a parallel mechanism, redox balancing helps in activating different hormones in a co-ordianted manner, which maximizes the positive impact of TU supplementation. The ABA level was regulated temporally which facilitated the induction of jasmonate to prioritize defence over growth in NaCl + TU treatment. In contrast, ABA level was consistently high under NaCl and hence, jasmonate increase was not seen. The auxin level was also higher in NaCl + TU than NaCl which supports root growth. The cumulative impact of miRNA and hormone mediated changes were reflected in terms of improved plant growth under NaCl + TU as compared with NaCl treatment.

In conclusion, present study utilized the exogenous application of TU, a non-physiological thiol and ROS scavenger, to develop redox regulated miRNA and hormone based regulatory module operative under NaCl stress condition (Fig. 6). The supplementation of TU modulates post-transcriptional gene regulation to enhance the expression of salt tolerance related genes. This is mediated either through down-regulation of miRNA expression or via one of the possible mechanisms of decoupling miRNA:mRNA inverse relationship. Simultaneous to this, levels of different hormones were co-ordinated to maximize TU mediated ameliorative effect. ABA level was regulated temporally which facilitated the induction of jasmonate to prioritize defense over growth in NaCl + TU treatment. This was supported from higher level of auxin which reduced the toxicity on root growth. The overall effect was reflected in the form of improved growth phenotype. Thus, the study highlights the significance of redox homeostasis, as one of the “core” regulators, for mediating miRNA and hormone based regulation through a bifurcative mechanism. Additionally, the mechanistic basis for salt-ameliorating action of TU has also been provided which ensures wider adoptability of TU-based formulations for imparting crop resilience to salt stress under farmer’s field condition.

## Materials and Method

### Plant material, growth condition and stress treatments

The entire study was performed on Indian mustard (*Brassica juncea* var. TPM1). Equi-sized seeds were surface sterilized with 30% ethanol for 3 min and then washed thoroughly to remove traces of ethanol. The sterilized seeds were soaked in distilled water for 6 h and then uniformly spread on petri plate having wet cotton bed covered with Whatman filter paper No. 3. Following 22–24 h of incubation under dark, seeds were planted on a customized circular thermocol disc having 18 equally spaced holes to hold seedlings. The bottom of the disc was tightly covered with 2 pieces of net with a very thin layer of absorbent cotton in between. From the top of disc, 0.4% agarose was filled in each hole to avoid any drying of the seedlings. The germinated seeds were planted on these discs and then were floated in 1 L beaker containing 800 ml of medium, covered with black paper till 800 ml mark to avoid any hindrance in root growth due to light. The hydroponic set-up was shifted in growth chamber (Sanyo, Japan) having a daily cycle of a 14 h photoperiod with a light intensity of 150 μE m^−2^ s^−1^, day/night temperature of 25/22 °C and relative humidity of 65–75%. The constant ratio between plant number and medium volume was maintained throughout the study.

To observe the ameliorative potential of TU under NaCl stress, post-germination phenotyping was performed. During the entire course of phenotyping, a total of four hydroponics sets having three independent biological replicates in each set were grown under control condition for 10 d and were subsequently subjected to different treatments such as control (1/2 MS), NaCl (150 mM), NaCl (150 mM) + TU (75 μM) and TU (75μM). At every alternate day, the level of medium was maintained with respective solution. Differential phenotypes were recorded at 7d post treatment in terms of survival efficiency. In order to obtain leaf phenotype, NaCl concentration was reduced to 125 mM and phenotype was recorded at 10 d post treatment in terms of fresh weight per seedling and leaf area. For small RNA sequencing and real-time PCR, seedlings grown under control condition for 15 d and then were subjected to different treatments such as control (1/2 MS), NaCl (125 mM), NaCl (125 mM) + TU (75 μM) and TU (75 μM). At 4 h post treatment, root and shoot were harvested separately, snap chilled in liquid N_2_ and stored at −80°C. The estimation of different hormones and cations like sodium and potassium were performed at 24 h after treatment; expect for ABA which was estimated at both 4 and 24 h after treatment. DAB based staining was performed at 4 h after treatment. For NaCl + TU and TU alone treatments, 24 h pre-treatment with same concentration of TU was also given.

### Measurement of leaf area

The fully expanded first secondary leaves from each treatment were mounted on a transparency slide with 50% glycerol and then scanned using HP Scanjet G4010 flatbed scanner with a resolution of 300 dpi. Leaf area was calculated using LAMINA ver. 1.0.2^79^. The threshold was set to greedy search and all the check marks were set uncheck with rest default settings.

### DCFDA based histochemical imaging, RNA isolation

DCFDA based staining was performed as per the method described previously^26^. The total RNA was extracted using mirVANA kit (AM 1560; as per manufacturer’s instruction). RNA quality was assessed in terms of 260/280 and 260/230 ratios and intactness of rRNA bands on 1.2% denaturing agarose gel. The bioanalyzer profile was also generated and RIN number more than 8 was considered as good quality RNA for sequencing.

### Small RNA sequencing and data analysis

The entire workflow for isolating small RNA, making barcoded library and sequencing was performed on SOLiD platform ver 4.0 as per manufacturer’s instructions (Applied Biosystems). Raw de-condensed sequencing reads obtained from SOLiD sequencing platform were processed for adapter cleaning using primitive 3’-adapter cleaning with an alignment of minimum 8 bp adapter. The trimmed reads were filtered off for contamination from other nc-RNAs by aligning them against other ncRNAs, such as RFAM available from ftp://ftp.sanger.ac.uk/pub/databases/Rfam/10.1/, non-coding repeat library REPBASE available from http://www.girinst.org/repbase/update/index.html and genome predicted tRNA available from http://lowelab.ucsc.edu/tRNAscan-SE/. Comparative synteny analysis between *Brassica* lineage reveals the conserved linkage arrangements and collinear chromosome segments between *B. rapa* and *A. thaliana*, which diverged from a common ancestor approximately 13–17 million years ago^80^. Considering this relationship and since *B. juncea* (2n = 36, AABB) is an allotetraploid derived from interspecific hybridization between *B. rapa* (2n = 20, AA) and *B. nigra* (2n = 16, BB) followed by spontaneous chromosome doubling, we applied cross-species mapping approach for the identification of novel miRNAs. Following filtering, mapped reads were analyzed for the identification of miRNAs using two independent methods by comparative cross-mapping of reads to *Brassica rapa* Chiifu-401 v1.2 assembly available from Phytozome (http://www.phytozome.net) using bowtie and Shortstacks^81^ and using sRNAtoolbox^82^. For both novel and conserved miRNAs, 20–24 nt length threshold were kept. Subsequently, all putative hairpins and identified microRNAs were analyzed for structural analysis using RNAFold. In addition, read clustering approach^83^ was also implemented for the identification of the novel miRNAs. We clustered the reads mapping to identical positions and define those as “read clusters” in the following way: (i) reads were sorted by read count (read frequency); (ii) most frequent read was assigned to first read cluster (the coordinates of the read cluster are given by the coordinates of the most frequent read); (iii) for all other reads, we checked if the read lay within a window defined by ClusterStart –3’ nt and ClusterEnd + 5’ nt on the same strand (flanking were added in order to assign all isomiRs to the same read cluster); (iv) if the read belonged to an existing cluster, associated read information (sequence and the read count) was added to the cluster; and (v) if the read did not belong to an existing cluster, a new cluster was opened. After clustering all reads, we extracted pairs of read clusters with distances of less than 150 nt between each other, because for bona fide miRNAs there should be two read clusters corresponding to two arms processed from pre-miRNA sequence. For the identification of differentially expressed miRNAs, firstly, redundant miRNAs identified from two approaches were checked for redundancies and a non-redundant set of the miRNAs having the corresponding precursor miRNAs was obtained for expression profiling. Following, each library was mapped individually to non-redundant set of miRNAs and the read counts obtained were used for the identification of differentially expressed genes using EdgeR^84^, DESeq2^85^ available from Bioconductor (http://www.bioconductor.org). Based on FDR- and p-value, miRNAs exhibiting minimum 1-fold change with a significant p-value < 0.05 over control were considered as significantly up- or down-regulated.

### Identification of putative targets for conserved and novel miRNAs

For the entire set of differentially expressed miRNAs, putative targets were predicted using two approaches: 1. Previously published transcriptome of *Brassica juncea* [GSE6242^86^; GBEQ01^87^ and GSE73201^88^] and 2. In case of no targets identified in *Brassica juncea*, targets were identified using *Brassica rapa* as a reference genome. The psRNATarget with settings: Maximum expectation: 2.0, length for complementarity scoring (hspsize): 20, number of top target genes for each small RNA: 200, Target accessibility - allowed maximum energy to unpair the target site (UPE): 25, flanking length around target site for target accessibility analysis: 17 bp in upstream/13 bp in downstream, range of central mismatch leading to translational inhibition: 9–11 nt were used for target prediction. The target gene details are mentioned in Table. 1. To identify enriched GO categories, we primarily looked for the GO enrichment in case of targets in *Brassica juncea,* however due to the poor annotation of the assembled transcripts, no significant enrichment was seen. Then after, we did a target prediction of all differentially expressed miRNAs against *Brassica rapa*, taking into account the close genome synteny and gene order in Brassica species, and putative GO as per annotations were looked for enrichment using Fischer- exact test followed by Boneferroni correction with a p-value threshold of 0.05 (Supplementary Tables 12 and 13).

### Stem-loop and Quantitative real-time PCR for expression profiling of miRNAs their targets

Stem-loop PCR was performed as per protocol described previously^89^. The isolation of RNA and quantitative real-time PCR was performed as described previously^28^. Details of the transcript sequence used for designing primers can be found as Supplementary Information-1. Details of the primers used for novel miRNA stem-loop PCR and conserved and novel miRNA target can be found as Supplementary Tables 14 and 15, respectively.

### Quantification of cations and plant hormones

The sample preparation and estimation of cations were done as per protocol described previously^90^. For ABA estimation, samples (~200 mg fresh weight) were dissolved in MilliQ water (1:20 w/v) and then kept under shaking condition at 4 °C overnight. The content was centrifuged at 8500 g at 4 °C for 15 min and supernatant was used for ABA estimation using ABA detection kit (Agdia; PDK 09347), as per manufacturer’s protocol. For auxin and jasmonate, the extraction and analysis were performed as per the protocol described previously by^91^. The representative HPLC peak profile for auxin and jasmonic standards can be find as Supplementary Figures 4 and 5, respectively.

### Statistical analysis and sequencing data deposition

The entire experiment was carried out in a completely randomized design and repeated at least twice to check reproducibility. One–way analysis of variance (ANOVA) was done on all the data to confirm the variability. Duncan’s multiple range test (DMRT) was performed to determine the significant difference between treatments using statistical software SPSS 17.0. Sequencing reads corresponding to this study are available from EBI SRA under the project number PRJEB9362.

## Additional Information

**How to cite this article:** Srivastava, A. K. *et al*. Thiourea priming enhances salt tolerance through co-ordinated regulation of microRNAs and hormones in *Brassica juncea. Sci. Rep.* **7**, 45490; doi: 10.1038/srep45490 (2017).

**Publisher's note:** Springer Nature remains neutral with regard to jurisdictional claims in published maps and institutional affiliations.

## Supplementary Material

Supplementary Information

Supplementary Table 6

Supplementary Table 7

Supplementary Table 8

Supplementary Table 9

Supplementary Table 10

Supplementary Table 11

Supplementary Table 12

Supplementary Table 13

Supplementary Table 14,15

## Figures and Tables

**Table 1:** Target gene prediction of conserved and novel miRNAs showing differential expression in root and shoot.

miRNA	Expect Value	Accession		Annotation
		*B. juncea*	*B. rapa*	
Targets for differentially induced conserved miRNA in root and shoot				
bra-miR158-3p	2.5		Brara.I01252.1	PPR repeat containing protein
bra-miR161-5p	1.5	c127222_g1_i1		PPR repeat-containing protein
bra-miR171e	0	TCONS_00042811		Scarecrow-like protein 6
bra-miR390-3p	3		Brara.H02653.1	VACUOLAR PROTEIN SORTING-ASSOCIATED PROTEIN 18 HOMOLOG
bra-miR403-3p	2		Brara.I05028.1	YqaJ-like viral recombinase domain (YqaJ)
bra-miR824	0.5		Brara.K00360.1	AGAMOUS-LIKE MADS-BOX PROTEIN AGL17-RELATED
bra-miR157a	1		Brara.F03810.1	SQUAMOSA PROMOTER-BINDING-LIKE PROTEIN 10-RELATED
bra-miR164a	1	TCONS_00044570		NAC domain-containing protein 21/22
bra-miR172c-3p	0.5	TCONS_00050888		AP2-like transcriptional factor
bra-miR395-3p	1.5	c70764_g5_i1		ATP sulfurylase
bra-miR168b-5p	3		Brara.E01925.1	EUKARYOTIC TRANSLATION INITIATION FACTOR 2 C
bra-miR171a	1	TCONS_00056693		Scarecrow-like protein 15
bra-miR172d-3p	1	TCONS_00012951		AP2-like ethylene-responsive transcription factor TOE2
bra-miR390-5p	2	gi|674884630|emb|CDY47879.1|		Serine-type endopeptidase activity
bra-miR396b-5p	2	TCONS_00046314		Growth-regulating factor 1
bra-miR5654a	2.5		Brara.I05094.1	PPR repeat family protein
bra-miR1140	2.5		Brara.I04295.1	Pectate lyase superfamily protein
bra-miR172b-5p	3		Brara.G00265.1	ALUMINUM-ACTIVATED MALATE TRANSPORTER 4-RELATED
bra-miR1885b	0	TCONS_00055693		UDP-arabinose 4-epimerase
bra-miR319-5p	2	TCONS_00058075		Mechanosensitive ion channel domain-containing protein
bra-miR395-5p	1.5	gi|656686826|gb| GBEQ01070429.1		ATP sulfurylase
bra-miR158-5p	3		Brara.C04365.1	Arabidopsis protein of unknown function (DUF241)
bra-miR161-3p	2	TCONS_00015119		Pentatricopeptide repeat-containing protein
bra-miR391-3p	2	TCONS_00063918		Pentatricopeptide repeat-containing protein
bra-miR396-3p	2.5		Brara.I02082.1	PHOSPHATE TRANSPORTER PHO1 HOMOLOG 2-RELATED
bra-miR9555a-3p	3		Brara.B01903.1	Leucine-rich repeat transmembrane protein kinase
bra-miR164a	1	TCONS_00044570		NAC domain-containing protein 21/22
bra-miR395a-3p	1.5	c70764_g5_i1		ATP sulfurylase
bra-miR403-5p	2.5	Brara.I03870.1		Plant protein of unknown function (DUF827)
bra-miR5725	2	c68837_g4_i12		Kinase family protein
bra-miR172d-5p	3		Brara.D00057.1	Beta glucosidase 10
bra-miR160a-5p	0.5	c69810_g1_i2		Auxin response factor 17-2
bra-miR161-3p	2	TCONS_00015119		Pentatricopeptide repeat-containing protein
bra-miR5712	1.5	TCONS_00068909		cDREB
bra-miR9563a-5p	2	c60087_g2_i2		Calmodulin-domain protein kinase CDPK isoform 2
Targets for differentially induced novel miRNA in root and shoot				
bra-miRX1	2		Brara.H03095.1	Fzo-like
bra-miRX2	1.5		Brara.D02054.1	Pho2
bra-miRX3	2		Brara.G00856.1	Galactose Oxidase
bra-miRX4	1.5		Brara.B00890.1	Brevis Radix-4
bra-miRX5	1.5		Brara.K00675.1	CBL-10

The target genes for differentially induced conserved or novel miRNAs were predicted using transcriptome assembly of *Brassica juncea* or *Brassica rapa* genome (refer methodology for details regarding target prediction). The transcript sequences of all these targets used for primer designing are mentioned in Supplementary information-1.
