# Supplementary material for: Thiourea priming enhances salt tolerance through co-ordinated regulation of microRNAs and hormones in Brassica juncea
Source: Sci Rep. 2017 Apr 6;7:45490. doi: 10.1038/srep45490 (PMC5382540; doi:10.1038/srep45490)
Supplement: Supplementary Information [file srep45490-s1.pdf]

<u>**Supplementary Information**</u>

Thiourea priming enhances salt tolerance through co-ordinated regulation of microRNAs and hormones in *Brassica juncea*

Ashish Kumar Srivastava[1*], Gaurav Sablok[2], Michael Hackenberg[3], Uday Deshpande[4], Penna Suprasanna[1]

Supplementary Fig. 1: Read length distribution analysis: A) Distribution of adapter cleaned reads length across the libraries; B) Distribution of reads mapped in *Brassica rapa* genome across the libraries.

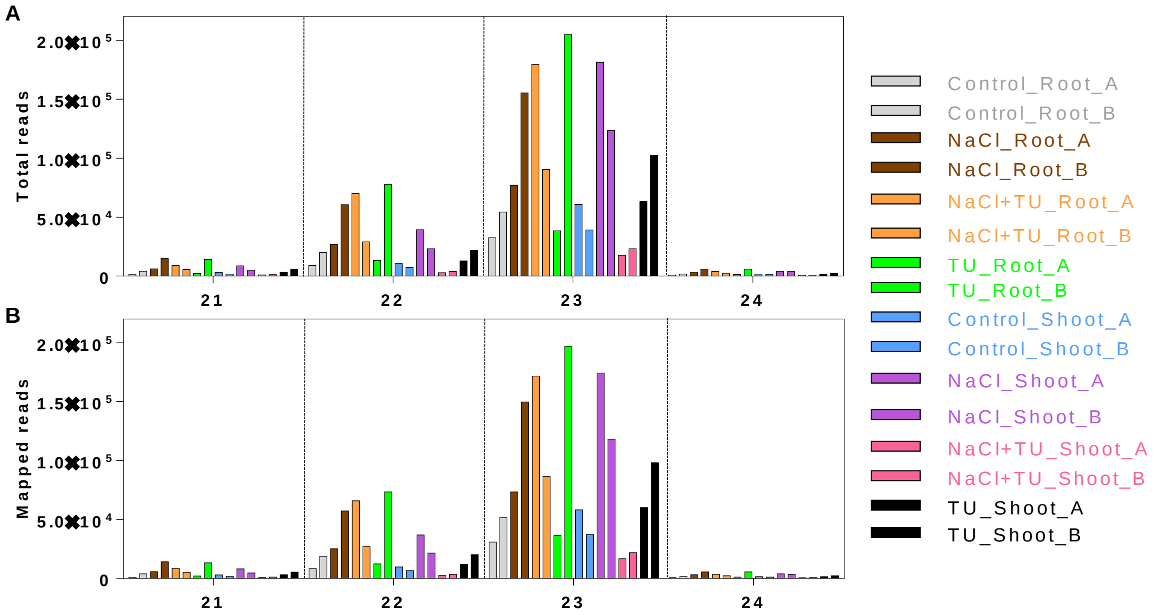

Supplementary Fig. 2: Heat-map showing the expression profile of conserved (A) and novel (B) miRNAs in root and shoot of *B. Juncea* seedlings under different treatments. The seedlings were grown hydroponically for 15 d and then subjected to different treatments such as control (1/2 MS), NaCl (125 mM), NaCl (125 mM)+TU (75 µM) and TU (75 µM). For NaCl+TU and TU alone treatments, 24 h pre-treatment with same concentration of TU was also given. At 4 h after treatment, roots were harvested independently and subjected to small RNA sequencing on SOLiD platform. The heatmap displays the normalized log2readcounts across the conditions.

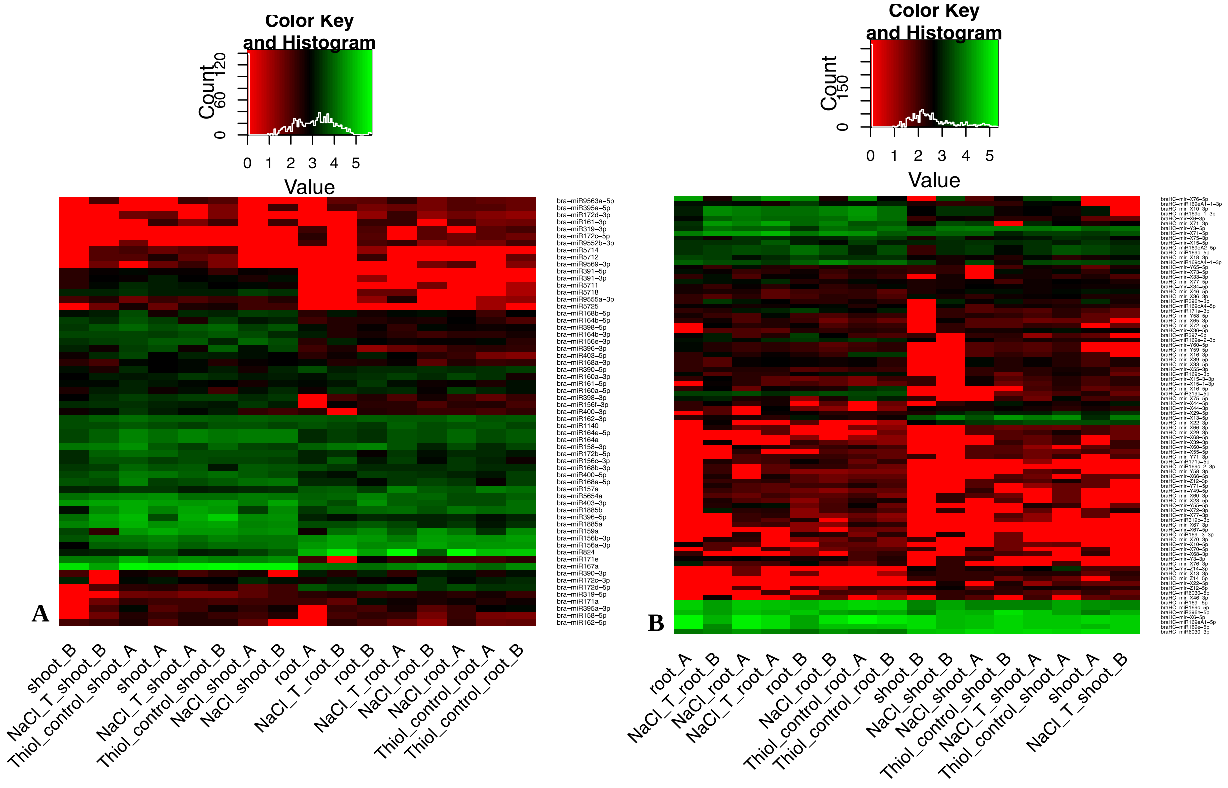

Supplementary Fig. 3: Melting curve profiles of novel miRNAs. The novel miRNAs were amplified from total RNA using stem-loop PCR (refer supplementary table-14 for primer details). The Corbett Rotor Gene 6000 machine was used for generating melting curves. The single peak on melt curve show specific amplification. The mature miRNA sequence was confirmed by sequencing amplified product of stem-loop PCR.

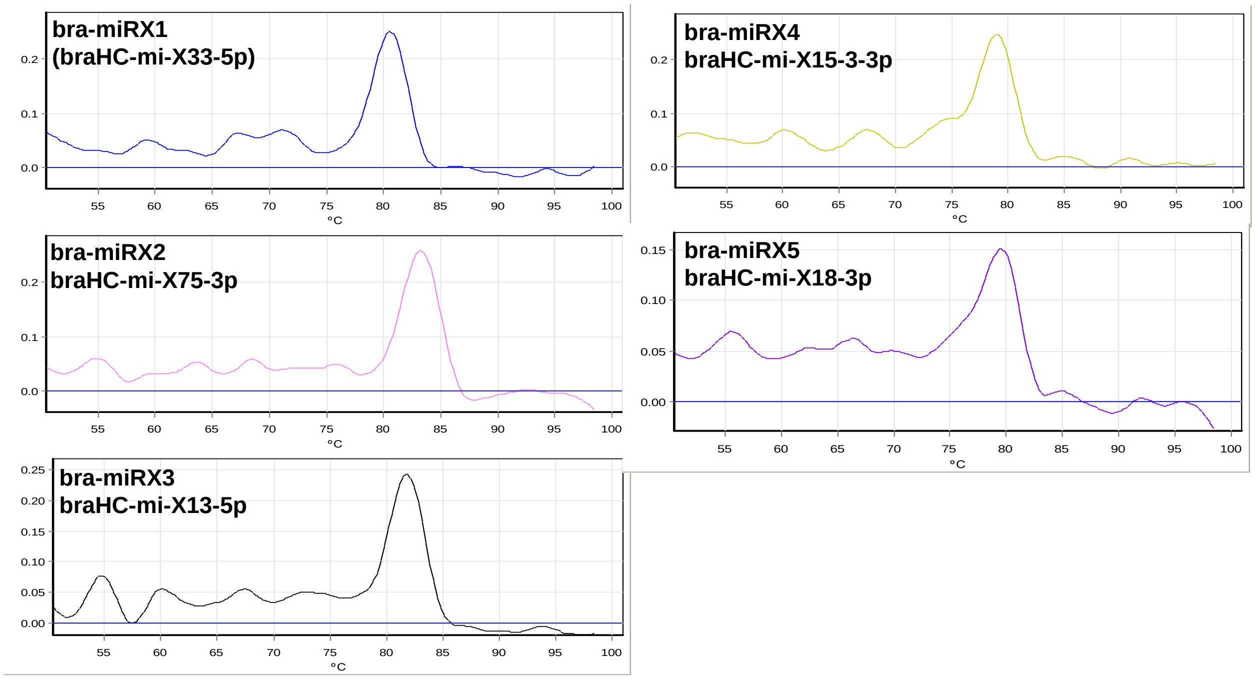

Supplementary Fig. 4: Representative HPLC profile showing the peak separation of auxin standards. The standards (10 mg/ml) were mixed together in 1:1 ratio to make 100 μl and then 25 μl of this was injected in HPLC to follow their separation as per the method described previously by Srivastava et al. (2013).

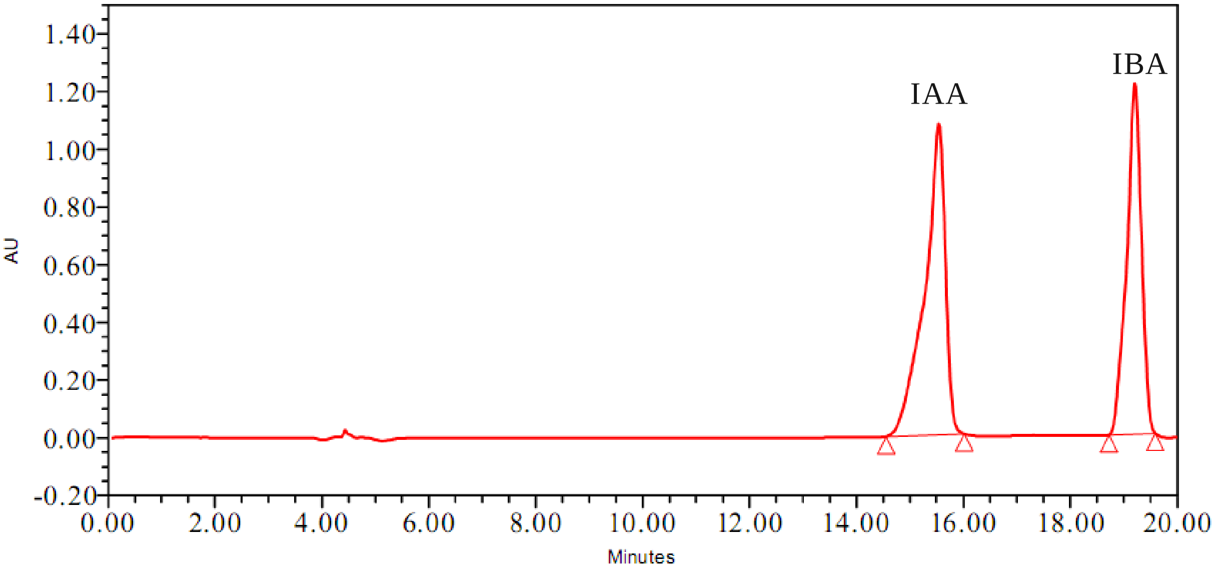

Supplementary Fig. 5: Representative HPLC profile showing the peak separation of jasmonate standards. The standards (10 mg/ml) were mixed together in 1:1 ratio to make 100 µl and then 50 µl of this was injected in HPLC to follow their separation as per the method described previously by Srivastava et al. (2013).

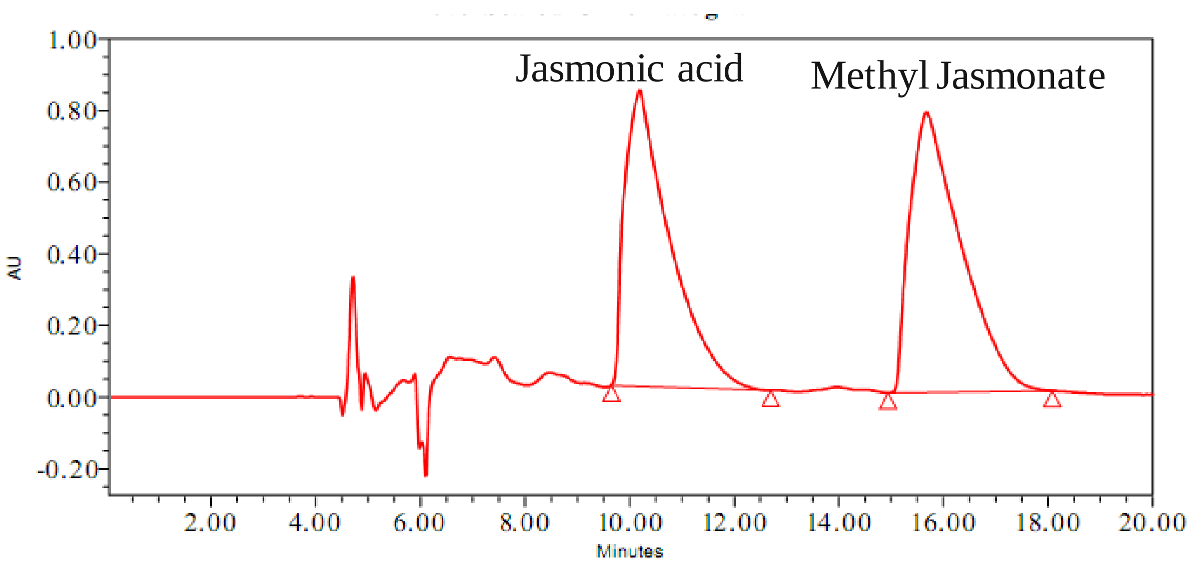

Supplementary Table- 1: Sequencing and mapping summary of the smallRNAs reads using cross species mapping to *Brassica rapa* genome.

Organ	Treatment	Raw Reads	Genome mapped reads	Unique mapped reads	Unique reads mapped to genome	Genome reads mapped (%)
Root	Control_A	91717	82067	59082	53694	89.48
	Control_B	503035	453057	331669	306487	90.06
	NaCl_A	260889	225762	137851	120401	86.54
	NaCl_B	690306	566362	316173	272650	82.05
	NaCl_TU_A	459802	406978	201884	180129	88.51
	NaCl_TU_B	298500	255444	105401	89938	85.58
	TU_A	1369302	1131501	581416	495788	82.63
	TU_B	1448849	1206182	678602	586740	83.25
Shoot	Control_A	337491	321244	100845	93983	95.19
	Control_B	120950	113734	39348	36247	94.03
	NaCl_A	380611	360055	76606	69911	94.60
	NaCl_B	175370	167902	44720	41012	95.74
	NaCl_TU_A	711388	668119	282595	265823	93.92
	NaCl_TU_B	351951	333526	108109	99923	94.76
	TU_A	462435	437111	190325	179353	94.52
	TU_B	351775	330183	163110	153080	93.86

Supplementary Table-2: Expression profiling of conserved miRNAs from *Brassica juncea* roots using small RNA sequencing. The *B. juncea* seedlings were grown hydroponically for 15 d and then subjected to different treatments such as control (1/2 MS), NaCl (125 mM), NaCl (125 mM) + TU (75 μM) and TU (75 μM). For NaCl+TU and TU alone treatments, 24 h pre-treatment with same concentration of TU was also given. At 4 h after treatment, roots were harvested independently and subjected to small RNA sequencing on SOLID platform. The differential expression is depicted either on the basis of their statistical significance (*) where $p<0.05$ or on the basis of their expression significance (**) where log fold change is ≥1. All the differentially expressed miRNAs are also marked as bold.

miRNAs	C Vs. NaCl	C Vs. NaCl+TU	C Vs. TU
**A. On the basis of statistical significance (logFC/p-value)**			
bra-miR158-3p	**-2.27/0.02***	**-1.20**/0.20**	**-1.54**/0.1**
bra-miR161-5p	**-1.91/0.02***	**-1.52**/0.08**	-0.93/0.27
bra-miR171e	-0.83/0.48	**-2.49*/0.04**	-0.53/0.66
bra-miR390-3p	-0.38/0.71	**-3.15*/0.003**	-0.35/0.73
bra-miR403-3p	**-1.63**/0.07**	**-2.17*/0.02**	-0.55/0.53
bra-miR824	**1.71**/0.10**	**2.28*/0.03**	**1.47**/0.15**
bra-miR157a	0.39/0.61	0.91/0.22	**2.02*/0.01**
bra-miR164a	**1.45**/0.06**	0.36/0.64	**1.95*/0.01**
bra-miR172c-3p	**1.75**/0.10**	0.31/0.77	**2.39*/0.03**
bra-miR395a-3p	0.04/0.80	**1.02**/0.20**	**3.55*/0.002**
**B. On the basis of expression significance (logFC)**			
bra-miR168b-5p	-0.38	**-1.04****	-0.13
bra-miR171a	-0.74	**-1.95****	-0.18
bra-miR172d-3p	0.6	**1.61****	0.76
bra-miR390-5p	-0.64	**-1.56****	0.5
bra-miR396b-5p	-0.11	**-1.09****	0.23
bra-miR5654a	-0.82	**-1.40****	-0.54
bra-miR1140	0.44	0.03	**1.05****
bra-miR172b-5p	0.52	0.77	**1.21****
bra-miR1885b	0.41	-0.03	**1.27****
bra-miR319-5p	0.7	0.05	**1.24****
bra-miR395a-5p	0.64	0.79	**1.48****
bra-miR158-5p	**-1.03****	-0.79	**-1.71****
bra-miR161-3p	**-1.46****	**-1.13****	**-1.13****
bra-miR1885a	**-1.43****	**-1.13****	**-1.05****
bra-miR391-3p	**-2.43****	**-2.19****	**-2.87****
bra-miR396-3p	**1.66****	**1.70****	**3.00****
bra-miR9555a-3p	**-2.42****	**2.57****	0.42

Supplementary Table-3: Expression profiling of conserved miRNAs from *Brassica juncea* shoot using small RNA sequencing. The *B. juncea* seedlings were grown hydroponically for 15 d and then subjected to different treatments such as control (1/2 MS), NaCl (125 mM), NaCl (125 mM) + TU (75 μM) and TU (75 μM). For NaCl+TU and TU alone treatments, 24 h pre-treatment with same concentration of TU was also given. At 4 h after treatment, shoots were harvested independently and subjected to small RNA sequencing on SOLID platform. The differential expression is depicted either on the basis of their statistical significance (*) where $p<0.05$ or on the basis of their expression significance (**) where log fold change is ≥1. All the differentially expressed miRNAs are also marked as bold.

**miRNAs**	**C Vs. NaCl**	**C Vs. NaCl+TU**	**C Vs. TU**
**A. On the basis of statistical significance (logFC /p-value)**			
bra-miR164b-5p	**-1.64**/0.06**	**-1.73*/0.05***	-0.11/0.88
bra-miR172c-3p	-0.18/0.89	**-2.17*/0.04***	**-1.31**/0.22**
bra-miR396-3p	-0.61/0.54	**-2.72*/0.01**	0.65/0.48
bra-miR395a-3p	**2.06**/0.13**	**1.08**/0.38**	**2.93*/0.01**
bra-miR403-5p	0.26/0.85	**1.53**/0.07**	**1.70*/0.05**
bra-miR5725	**1.62**/0.68**	**2.67**/0.14**	**3.31*/0.01**
bra-miR396b-5p	**1.43**/0.09**	**2.57**/0.001**	**3.32**/0.002**
**B. On the basis of expression significance (logFC)**			
bra-miR158-3p	**-1.81****	-0.23	0.26
bra-miR172d-5p	**-1.05****	-0.62	0.3
bra-miR319-5p	**1.09****	-0.56	-0.15
bra-miR160a-5p	**1.55****	**1.40****	0.44
bra-miR161a-3p	**-1.08****	**-2.30****	-0.51
bra-miR172d-3p	**-2.20****	**-3.16****	-0.15
bra-miR395a-5p	**2.44****	0	**1.95****
bra-miR5712	**-4.44****	**-1.39****	**-2.00****
bra-miR1885b	0.18	**1.95****	**1.86****
bra-miR9563a-5p	0	**5.03****	**3.28****

Supplementary Table 4: Details of the mature and hairpin sequence of novel miRNAs.

microRNA	Mature miRNA sequence	Hairpin sequence
(bra-miRX-1) braHC-mir-X33-5p	GTTTTCAGGTGATTTTAGGGTG<br>T	TTAAAGTTTTCAGGTGATTTTAGGG<br>TGTTTAGGTGGAGTTTCTTAGTTAA<br>AAAAAAATTAAAACCCAAATCTCA<br>TGGTTTTAAGTTATATTCTAGAGTG<br>GTTTAACATAAATCACCTAAATCT<br>CTGCAACTTATTAA
(bra-miRX-2) braHC-mir-X75-3p	TGCCAAAGGAGAGTTGCCCTG	AATAGGGCATCTTTCTATTGGCAC<br>GAGACAGTGAGATGTGCTCTTTTTT<br>TTGTGTGTGTTCATGGCTACTTGGC<br>TCTGTCACTTGCCAAAGGAGAGTT<br>GCCCTGTCACT
(bra-miRX-3) braHC-mir-X13-5p	TCTTTGGCATTCTGTCCACCT	TATCTTTGGCATTCTGTCCACCTCC<br>TCTACATATGTGTATGGACGATGT<br>GTTCTGTGAGTGGAGGTGGGCATA<br>CTGCCAATAGAGATCT
(bra-miRX-4) braHC-mir-X15-3-3p	TTTTTAGTTAAAAGTTAAGGGA<br>C	GGTTCCCTTAACTTTTAACTAAAAA<br>CGCTAAGAGATTTTTCTCAAGTCCC<br>TTATTTAAAGACCGTTCCTTAGTTT<br>TTTTAGTTAAAAGTTAAGGGACTC<br>TAC
(bra-miRX-5) braHC-mir-X18-3p	AATCGGACCAATAAAAAACTC	TTTTTGAGTTTTTTATTGGTCTGAT<br>TTATTTAATAATCGGTCGCCTAATC<br>GGTAAATGAGCCGATTAATCGGTT<br>ATTGGGCCGATTAATTGGTGACCG<br>ATTTTCTGGTCCGATTATGGTAAAT<br>TCGGGGCGGTCGGTCTTTATAATA<br>AATCGGACCAATAAAAAACTCAAA<br>AA

Supplementary Table-5: Stem-loop real-time PCR based expression profiling of novel miRNAs from root and shoot of *Brassica juncea*. The stem-loop PCR based expression profiling of identified novel mirNAs were performed from root (A) and shoot (B) of seedlings given different treatment conditions such control (1/2 MS), NaCl (125 mM), NaCl (125 mM) + TU (75 μM) and TU (75 μM) for 4 h. For NaCl+TU and TU alone treatments, 24 h pre-treatment with same concentration of TU was also given. The values represent the average $\log_2$ value of expression fold different of three independent biological replicates. The change in log2 expression ≥1 is given bold and marked with asterisk (*).

**A. At 4 h after treatment in Roots**				
**S. No.**	**miRNAs**	**logFC/SD as per stem-loop PCR**		
		**C Vs. NaCl**	**C Vs. NaCl+TU**	**C Vs. TU**
1.	braHC-mir-X33-5p (bra-mirX1)	-0.022/0.003	**-1.062/0.036**	-0.71/0.034
2.	braHC-mir-X75-3p (bra-mirX2)	0.276/0.025	**-1.862/0.023**	**-5.36/0.032**
3.	braHC-mir-X13-5p (bra-mirX3)	0.41/0.080	**-5.362/0.09**	**-5.983/0.078**
4.	braHC-mir-X15-3-3p (bra-mirX4)	0.442/0.053	-0.276/0.010	0.695/0.062
5.	braHC-mir-X18-3p (bra-mirX5)	0.313/0.023	**-1.589/0.015**	-0.66/0.036

**B. At 4 h after treatment in Shoot**				
S. No.	**miRNAs**	**logFC/SD as per stem-loop PCR**		
		**C Vs. NaCl**	**CVs. NaCl+TU**	**C Vs. TU**
1.	braHC-mir-X33-5p (bra-mirX1)	0.832/0.157	**1.668/0.328**	0.185/0.040
2.	braHC-mir-X75-3p (bra-mirX2)	0.714/0.071	**1.537/0.151**	-0.095/0.013
3.	braHC-mir-X13-5p (bra-mirX3)	0.705/0.122	**1.165/0.258**	-0.932/0.055
4.	braHC-mir-X15-3-3p (bra-mirX4)	0.543/0.096	0.445/0.031	-0.539/0.029
5.	braHC-mir-X18-3p (bra-mirX5)	**1.021/0.116**	**1.113/0.050**	0.346/0.044

Supplementary Information-1: Transcript IDs and sequences of all the microRNA targets whose expressions are validated using real-time PCR. For details of the primer sequence refer Supplementary Table-15.

1) bra-miR158-3p (Brara.I01252.1; Pentatricopeptide repeat (PPR) superfamily protein)

ATGTTGGCTAGGGTTTGCAGATCCAAATCTTCTTCTCCTGCTGTTTCAGCGGCTAGATTGTT
CTGTACGAGATCGATTCGTCATGCTCTGGCCAAGGAAAGTAGTGTTGGAGAGAGTGGGGG
AAGCAGGAGCGGGTTTCATGAAATCAAAGGGTTAGATGATGCGATTGATTTGTTCAAAGAC
ATGGTACGATCTAGTCCTTTACCTTCAGCAATCGATTTCAACAAGCTAATGGGTGTGGTGG
TGAGAATGGAAAGGCCCGATCTTGTGATTTCTCTCTATCAGAAGATGGAAAGGAAACAGAT
TCGACGTGATATCTACAGCATGAACATCCTTATAAAATGTTTCTGCAGCTGCTCTAAGCTTC
CCTTTGCTTTGTCTACATTTGGATCAGTGATAAGTATCTTCATAGACGGATATTGTGGGGCT
AAGAGGGTAGATGATGGAATGGAACTTCTCCATGAGATGTCTAGAAGAGGATTAGTTGCTA
ACACAATTACTTACACCACTCTTATTCACGGGTTCTGTCAGGAGATGATTTCTAGTGGTGTG
TGCCTTAATGTCGTTACTTGTAGCACTTTACTGGACGGTATCTGCGATAATGGGAAACTAAA
AGACGCATTGGAAATGTTTAAGGTTATGCAGAAGAGTAAGATGGATCTTGATGCTAGTCAC
CCCTTCAACGCTGTGGAACCTGATGTTCAAACTTACAATATATTGATCTGCGGCTTGATCAA
TAACGGGAATTTTTTAGAGGCTGAGGAATTATATGAGGAGATGCCCCACAGAGGAATGGTT
GATGATGGGGTGGAGCTTTTCTGCGAGATGGGTCCAAGAGGGATAGTTGCTAATGCAATT
ACTTACATCACTTTGATTCGTGGTTTTGGTAAAGTGGGTAATATTAATGGTGCTCTAGACAA
TTTCCAGGAGATGATTTCAAGTGGTGTAATTCCTGATACCATTACTATCCGCAATATGCTGA
CTAGTTTATGGAGTAAAGAGGAACTAAAAAGAGCAGTGGCAATGCTTGAGGATCTGCAGAT
GAGTATGTTATATTATTGGTCTGAACTAAAGAAGCACACCTTCCAGAAGATTTCAGGTGTTA
AAAGATGTTTAGGTGTCTTTCCTTTCTGTAGCTGTCTCCATGGTTATTGTCAAGCTCGGTCT
TCATGGTGGCTCCAAGGTTGGATCCAGAGAGAGTGA

2) bra-miR161-5p (c127222_g1_i1: gi|297801450|ref|XP_002868609.1| Pentatricopeptide repeat-containing protein)

ACGGACTTGTGGATAACGCGATGAGCTTGTTTAACCAAATGGAGATTAAAGCTGACGCCGT
TACTTACACGTCTCTCGTGAACGGTCTTTGTAACTCCGGTAGGTTGAGTGACGCCGTTAGT
TTACTTAACGATATGATGAGAAGGAGGATCAACCCTGATGTAGTCACTTTCAATGCGTTGAT
CGACGGGTTTGTGAAAAAGGGGAAGCTTCTGGAGGCTAGAGAGGTCTACAGCGAGATGAT
TCGAGTCTCTGTAGCTCCTGATGTTTTCA

3) bra-miR171e
(TCONS_00042811: gi|15236725|ref|NP_191926.1| Scarecrow-like protein 6)

ACACAAAAGAGGAGAGAGAGAGAGAGAGAGAGGATGAAGCGCACTTTGCCACTTTAATAT
CCTGCACGCATCCACAACCCAGAAGAAGAAGAAGAAGAAGAAAAAGGACAGAAGAGACTT
GAGAAGATAGTAGTGAGCCAAAGGGTTTGTGAACTTTGTTTGTGCTTNNNNNNNNNNNNN
NNNNNNNNNNNNNNNNNNNNNNNNNNNNNNNNNNNNNNNNNNNNNNNNNNNNNNNNNNNN
NNNNNNNNNNNNNNNNNNNNNNNNNNNNNNNNNNNCAAAGAGCCCACCTCTGTTCTCAGC
CCCCTCACTACATCCTCCTCCACGGTGTCTTCCTCGCACGGCGTAAGCACTGCCGTCGGA
GGCGGCGGTATCTCCACTGCCATCTCCGGCGGCGNNNNNNNNNNNNNNNNNNNNNNNNNN
NNNNNNNNNNNNNNNNNNNNNNNNNNNNNNNNNNNNNNNNNNNNNNNNNNNNNNNNNNNN
NNNNNNNNNNNNNNNNNNNNNNNNNNNNGACAACGACCAGAGCATCCTCAGGCTCATCAT
GATGGGTGACTCCCAAGAAGATCCTTCTCACGAACTTAACAACATTCTCCAGACCTCATCA
GCTTCTCAATCTCCCGCCACCTTTCATAACTCCGACTACACATCCCTCGCCAGCTTCGGAG
TCGTCGATACCACCGGCTTCGGCTTAGACCACTCGGTCCCACCACCTCTGTTTCAGGCAC
AAGTCGAGGAGAAGCCGCCCCACCAACTCCTGATCAACCAAAGCCAAACTCATTTCACCCA
GAACCCCTCCCTCTTCTACGGCCATCATCATATCCCCGCGGCGGCGACGCTACCTCCGCC
GGCAAAGCGTCTCAACCAAGGCTCCGTGGGTCAAGGGATGATAACGGAGCAGCTGTTCAA
GGTGGCGGAGGTGATAGAGAGCGGTGGCGACACTAGTCTGGCTCAGGGGATATTGGCGC
GGCTCAATCAACAGCTCTCTTCTCCCGTGGGAAAGCCATTCGAAAGGGCAGCTTTCTACTT
CATAGAGGCTCTCCACAACTGTCTCCTCCACAACGGAAACGCCTCCCAACCACCCCTAAAC
CCTTACTCCCTCATCTTCAAGATCTCCGCCTACAAATCCNNNNNNNNNNNNNNNNNNNNN
NNNNNNNNNNNNNNNNNNNNNNNNNNNNNNNNNNNNNNNNNNNNNNNNNNNNNNNNNNNN
NNNNNNNNNNNNNNTGGCTTCAACCGTCTCCACATCATCGACTTCGACATCGGCTACGGT
GGCCAATGGGCTTCCCTCATGCAAGAGCTCGTCCTCCGCGACAACAACGCTCATCCCCTC
TCTCTCAAAATCACAGTCTTCGCTTCTCACGATGACCACCACCAGCAGCAGCTTGAGCTGG
GCTTCACTCAAGACAACCTCAAGCACTTCGCCTCGGAGATCAACATCTCCCTCGACATCCA
GGTTCTGAGCTTGGACCTCCTCGCCTCTTTCTCCTCCCCTAACTCCTCAGACAAAGAAGAA
GCCGTCGCCGTTAATCTCTCCCCCGCCTCCTTCNNNNNNNNNNNNNNNNNNNNNNNNNNN
NNNNNNTCCCTTCGCTCGTCCTCCGTTTCGTGAAACATCTCTCTCCGACGATCATCGTCTG
CTCCGACAGAGGGTGCGAGAGGACGGATCTGCCTTTCCCTCAGCAGCTCCTCCACTCGCT
GCAGTCACACGCCGCTCTCCTCGAGTCCCTCGACGCCGTCAACGCCAACCTCGACGCTAT
GCAGAAGATCGAGAGGTTTCTCATACAGCCGGAGATCGAGAAGCTCGTCCTCGATCGTAG
CCGTCCGATCGAAAGGCCAATGATGACGTGGCAAGCAATGTTCATGCAGATGGGGTTCTC
TCCTGTGGCCCACAGCAACTTCACGGAGTCTCAGGCAGAGTGTTTGGTTCAACGGNNNNN
NNNNNNACGCCGGTAAGAGGCTTTCACGTCGAGAAGAAACACAACTCTCTTCTTCTCTGCT
GGCAAAGGACAGAACTTGTCGGAGTCTCCGCCTGGAGATGTCGCTCCTGACTCATCCCTC
CCCA

4) bra-miR390-3p
(Brara.H02653.1; VACUOLAR PROTEIN SORTING-ASSOCIATED PROTEIN 18 HOMOLOG)

ATGGATCAAGGAAGGCAAGTGTTTGCAGTGGATCTTCTCGAGAGATATGCCACAAAGAATC
GTGGCATGATCACATGTATGGCTTCTGGAAACGATGTGATTGTTCTTGGAACAAGCAAAGG
ATGGATCATCCGCCACGATTTTGGACTTGGGAGTTCTTATGATATTGATCTCTCTGTTGGTC
GAACTGGGGACCAATCGATCCACAAGGTTTTTGTTGATCCTGGTGGTAGCCATTGCGTTGC
TACGGTGACTGGTGTTGGAGGAGCTGAGACTTTTTACACTCATGCTAAATGGCCTAAGCCG
CGTGTCTTGAGCCGTTTGAAAGGCTTGCTGGTTAACGCGGTGGCTTGGAACAAGCAGCAG
ATAACAGAAGTTTCAACTAAGGAGATCATCCTTGGCACTCAGGATGGGCAGCTGTTTGAGA
TGGCTGTCGATGAGAAGGATAAGAGAGAGAAGTACGTCAAGTTTTTGTTCGAACTTGAAGA
ACTTCCCGAGGCCTTCATGGATTTACAGATGGAAACAGCCAACATAAGCAGTGGAATGAGG
TACTATGTGATGGCTGTAACTCCTACTCGACTCTATTCCTTCACCGGGATTGGAACATTAGA
ATCTGTCTTTGCTAGTTATAAAGAACGTGCAGTTCGTTTTATGGAACTTCCTGGTGAAATAC
CAAACAGGCAAGTGCTAATCACCTTATATGAAAGAAATCACAAAGCTGAACTACATTTCTTT
ATAAAGCAACGAAGAGCGGTGCATTTTGCATGGCTTTCAGGAACAGGAATTTATCATGGTG
GCCTAAATTTTGGAGCTCAGCACAGTAATCCAAATGGTGATGAGAACTTTGTGGAGAGCAA
AGCGCTGTTGGACTACTCAAAACTGAGTGATGGCACTGAGGTAGTCAAGCCTAGTTCAATG
GCACTCTCAGAGTATCATTTCTTGCTTCTTATTGGGAATAAGGTTAAGGTTGTGAATCGGAT
AAGTGAACAAATCATAGAAGAGCTCCATTTTGATATATCGGCTGATTCAGCTTCGAGGGGC
ATCATAGGACTTTGCAGCGATGCCTCTGCTGGCCTTTTCTATGCGTATGATCAGAATTCGAT
CTTCCAGGTTTCTGTAATTGACGAAAGCAGAGATATGTGGAAGGTTCACCTAGACTTGAAA
GATTATGCTGCAGCTTTAGCAAACTGTCGTGACCCTCTCCAGAGAGACCAAGTTTATTTAGT
TCAGGCAGAAGCTGCATTTGCCAACAAGGAATATCTACGAGCAGCATCATTCTATGCCAAA
GTCAATTATGTAATATCTTTTGAAGAGGTCACCTTGAAGTTTATTAGTATAAACGAACTGGAA
TCTTTGAGAACATTCCTGTTGCGAAAGCTTGATACTCTCTCAAAGAACGATAAATGCCAAAT
TACGATGATATCAACATGGGCAACAGAGCTATATCTGGACAAGATAAATCGTCTGCTTTTG
GAAGATGACACTGCTATAGAAAATCGTAACTCAGAGTATCACTCAGTCATTCAAGAATTCCA
CTAA

5) bra-miR403-3p
(Brara.I05028.1; YqaJ-like viral recombinase domain (YqaJ))

ATGAGCTATTCATTTACAGAGGCATCTTGTATGCAACATTGGCGTAAAAACTGGGACCACC
AAAGAAAGAACCGATTAACATCGAGCACATTCGCTCAAGCCATCGGGTTCTGGCCAAACCG
TAGAGTCCAGCTCTGGTTTGAGAAAATCGGAGCGATAGAGCCGTTCTCAGGCAACGCAGC
CACGTGCTGGACCAAGATCAAAGAGCTAGAAGCGCTCAACCGATACCACGTTCTAACGGG
ACACGACTTCGTCTTTCCCGAGTTCGTTACTTTAACAGAAGACGAAAACTGGTTAGGAGCT
TCTCCCGACGGAGACATGTACGGTTTGGTCAGCGAGGGGTCGAAAGGTATGCTGGAAGTT
AAGTGTCCGTACGGAGAGGGTTACCCGTGGAAGAAAGTGCCGTGGCATTACGTGCCGCA
GGCTCAGGGTTTGATGGAGATTGTGGGGAGAGATTGGTTGGATTTGTATTGTTGGACTGTT
AACGGGAGTAGTTTGTTTAGGATTGAGAGAGATTGTGTGTTTTGGCGGGAGATGAAACCGA
CGCTTGTTGATTTTTGGGAGAGGCATGTGGTTCCCAGGTTTAGTGATAGCTCTCTTGTGAT
AACAGATCCTTTGGTGGAGCTGGTGGAGTTTGTGCCTGAGTCTAGACATGAACGGTGTAAT
CAGATTTTGCGTGGGACTGAGAGGGTTATGGATAACTCTTGTAAGCGTTTGTTTTATGAAAT
TAATGGCCAAATCTTGGACTGA

6) bra-miR824
(Brara.K00360.1; AGAMOUS-like 16)

ATGGGAAGGGGAAAGATCGCGATTAAGAGGATCGATAACTCGACGAGTCGTCAGGTGACT
TTCTCCAAGAGAAGGAACGGCTTGTTGAAGAAAGCCAAGGAGCTTGCGATTCTTTGCGATG
CTGAAGTTGGTGTTATCATCTTCTCTAGTACTGGCAGGCTCTACGATTTCTCCAGCTCCAG
CATGAAATCGGTAATAGAGAGATACAGAGATGCCAAATGTGACACCAATTCGGAAATGAAC
CCAGCTTCAGAACTCAAGTTTTGGCAAAACGAGGCTGCGATTCTAAAGCGTCAGCTACATA
ACTTGCAAGAAAACCACCGGCAAATGATGGGTGAAGAGCTCTCTGGACTAAGTGTGGAAG
ATCTTCAGAAACTGGAGAATCAGCTTGAGATGAGCCTTCGTGACGTACGAATGAAAAAGGA
ACAAATGTTAGTCGAAGAAATAAAAGAACTGAACCGAGAGGGTAATCTCGTGCACCAAGAG
AATTTAGAGCTCCATAAGAAAGTAAACCTAATGCACCAGCAGCACATGGAGTTACATAAGA
AGGTTTCAGAGGTTGAGAGTGTGAAAAGCGCAGACAAAATTTCTCTTCTCACAAATGGTCT
AGACATGGGAGGTAACTCGAGCGAACATGTCCATCTTCAGCTCAGCCAACCCCAGCAGCA
TGACGAAACAAGTTCCAAAGCTATCCAACTAAGCTATTTTTCTTTCATTGCGTGA

7) bra-miR157a
(Brara.F03810.1; Squamosa promoter binding protein-like 2)

ACGCTCTTTGATCTACCAAAACATCCACATGTTTGTGTAACTACATATCAAATTGCTTTTTCT
TAATTAATAATATCATAAAGTTTCTTTACAAATAATAAAATCATGATTTTTCCTCAATAACGAA
TGAATCGTCCAAAAAAGTTGAAGCTGGTGAAAGGCAGTTTGACCCCTCCCCATCTCCCAAC
TTGGTACTTCGCTTTGCACACATCACATGCTTCCTCTCACTCTCTCTCCTCTTTTCTTCTCCC
ACTCTTGTTCTTCTCTCTAAATGCTCTGTACTGTGTCAAGCCAAAAATGGAAGCTTTACTCC
ATTAACGACGAGATCCTTCCCTAATTCTCTAATCAATAGGTAACCCATTTTTATTTAGACTAA
TCCTCATAACGCTTTTTGGTTTCCCGTAAATCTTATAGCTTAGAGTAAAGATTTTTATTTACT
TAGCTAAAAAGAAAGAACTTATTTACCATAAATGGTGAGACATCCATTTGCGGCTGAGATTT
TGCTAACTTTATTATCTCTCTCTTTCTTGATTTTTCTATTTTTCTTTTTTACTACCTACACAATA
GCATCATAATCAGAGAGGGATGGTGAGTTGAAAATGACCATTGTGTTGCATCTAGTTGATA
AATCTAAAAACCCTCAAATACCCCCACTTTCTCAAACGAAGTTAAAACAAATTTTACTCTTTT
ACTTTAGCCCTATAGATCTCATTCCCAAAGAAAAAAAAAAAAAAGAAAACCTTTTGTGGGTTG
TTGATGCTTTTTTGGTTCATGATCTCTTATGGGTTTTGTAAGTTACTGATTCTAGTGTCGGTT
TACATATGTGTTTAGGAACCATGGAATCTTTAGTGGGTTTGTGAGTAGTAGACAAATTTTAC
GAAATGTAATATTAAAGGGGATGATTTTGACAGCTATTGAGTAGTTAATCCGCATCTATGGA
CAGCTTTTGATAATGAAATGGTAAAGGAGGGTCCCTACTTCTTGAAGGATTATGCCCCCAC
AAACTCAATGAATGTAAATTATTGGTGAGCTAAAAGTTTTTTTTTTTGTGATGAAAAAAATGA
GGGTGTCTTTTATTGTTCTTGTTATAAATGAGAATGAAACTTGTGTTGGAAAGTCAAATAAAT
TTCTTCTTCCTCAGGGTTCACCTAAGAGAGAGATCCACAAGTTTTTTTAGATTTTGGTGTTTT
AGTGTCTGTATGGAGTGTAATGCAAAGCCATCATTGCAGTGGGAGTGGGATAATCTAATAT
CTTTTGGTACTTCATCAGCTGAAAATCTTAGAAAGCAACGACCAATGGATTGGGAAAATGAT
GGGTTTGATTGCACCACTTTTTACTCGTCCAGCTTTGCTACAGAAGCAGCTTATGGTGGTG
GTAGTTCAGGTTCTGATCTAGCTCATGCTTTCTCTAAAAGCTCAAAGTCAACTTCCATTAGC
TCTTCATCAGCTGAAGTGAGAACCTACAATTTCACATCAGAAGCTGGTGAAAGTGTTCCTC
CTGGAGAATTGGGGAGCAGTGAAGAGTTTGCAATGGGAATTGATACTTCTCCAAGTCTTGA
ACTCTCCTTCGGCTCTGGTGATCCGGTTCTTGGTTTGAAGCTTGGTAAGAGGACATACTTT
GAAGACTTTTGGGAAGTGGAGAATGCTAAAGGTTCAGCACTTCCGGTGAGCCTGGCGTCA
TCTTCTGCTTCTCCGGTGAAGAAATCCAAAACACTCTCTCAGAAATTACAAACTCCTCACTG
CCAAGTTGAAGGCTGTAACCTCGATCTCTCCTCAGCTAAGGACTATCATCGGAAACACAGG
ATTTGTGAAAACCATTCAAAGTTCCCTAAAGTCGTTGTAAGTGGAGTAGAGCGCCGGTTCT
GCCAACAATGTAGCAGGTTTCACTGTCTCTCTGAGTTTGATGAGAAGAAACGTAGCTGTCG
CCGGCGTCTCTCAGATCACAATGCAAGACGTCGCAAGCCAAATCCCGGGAGGACATATGA
TGGGAAGCAACAGATGGATTTTGTATGGAACAGATTTGCACTTATCCATCCAAGAAGTGAA
GAAAATTTTCTGTGGCCTAATCCGAAGCCTGTACCATCAAGAGGGTTATTGCATGAACCTG
CAAAGACCGAGATGCCCAATAAGCTTTTCACCGAGCATTGTGGATTTGGATTGTTGGACCC
CAAAACGAAAACCACCAGAGCTGAGTTATTCAGTAAAGATTTGTTAGAAAAGGTCACAATCT
CTTCTTCACACATGGGTGCTTCTCAAGATCTTGATGGTGCTCTCTCTCTTCTGTCAAATTCA
ACACCATGGGTTTCCTCGAACCAGCCAACACGGTTTTCCCTTAACCACCATTCCACAAGCA
ACCTCCAACCCGTGGTTCACGGGTCTGTGACTCAACTCAGTTCAGTGTCCAGCTACTGGCA

GCCGGACCCACCAGCAGCTGAGGGCTCAACCGCTTTGACTAGGAATGGGGTAGGCCAGT
TTAATGAGAACTACAACTTGAATCAGTTTTATAACTGAAAGTGTGTTGCTTTTAAAATCATAA
TAAGATACTTTGTGTAAGGATCAGGTGAGCCTAGTGGTAACGTTTAAATAGGAGCTGTGAA
ACTTGCTAAAGACATCAACTCCTCTCGTCTTTCTTTTGTCCATTGATTTTCTTGGGTTAGGG
AGAGTTGTGACAGTTAAGTATTATAAATCATCTTGTCAAATTATTTATACATTAGAATTTTCA
GATATTGATGGTGTAGCAACTGAAAAATT

8) bra-miR164a/164e-5p
(TCONS_00044570: gi|15222818|ref|NP_175997.1| NAC domain-containing protein 21/22)
TTTCTCTCTCTTTCCCTCTTAACTAATTGTCCGCTAATCAAAAAGATCATCATCAAATATTATA
AACCTAAAATCTAACCATTCAGTGATCAAAGGATCTCAACAAAGAGAGAGAGAGAGGAGCA
TGGAGTCGGAGGAGGAGAAGGAGAGTAGTATAAGCATGGTGGAGGCAAAGTTGCCCCCA
GGATTCAGATTTCACCCAAAGGACGACGAGCTTGTCTGCGATTACTTGATGGCANNNNNNN
NNNNNNNNNNNNNNNNNNNNNNNNNNNNNNNNNNNNNNNNNNNNNNNNNNNNNNNNNN
NNNNNNNNNNNNNNNNNNNNNNNNNNNNNNNNNNNNNNNNNNNNNNNNNNNNNNNNNN
NNNNNTGGCAAGGATTGGTATTTCTACAGCCAGAGAGATAGGAAATACGCGACTGGGCTG
AGAACAAACAGAGCAACGGCCACCGGATATTGGAAAGCCACAGGGAAAGACAGAGCCATT
CTAAGAAAGGGCAAGCTTGTTGGGATGAGGAAGACATTGGTGTTTTATCAAGGTCGAGCTC
CCCGTGGTCGTAAAACTGATTGGGTCATGCACGAGTTTCGTCTCCAGGGTTCCTTTGATCC
TCCTACTCTGAACTCTCCAGAGGTACAGGANNNNNNNNNNNNNNNNNNGGAAGACTGGGT
CCTGTGTAGAGTGTTCCACAAGAACACACAAGGAGACAACATGGGAAGCTGTTTTGACGA
GACAGTCTCCGCTTCTCTTCCTCCACTGATGGATTCTTACATCAACTTTGACCAAGAACNNN
NNNNNNNNNNNNNNNCCTCTTCTTACCTCGGTCATGATCAGCACTTCTTCACCAATGAGCA
AGTGCCCTGCTTCTCCAATTTGTCACAGAACCAAACCATACACTCAAACCTAACCGACTCA
GTCTCTGAACTCGAGACTCCTTGCAAGAACCCTATCCCCTTGTTCACTGGTGGTTCATCCC
CCGCCATGCTCCCAGGGCTAGATTCGTTCAGTTCATCAGATCAGATGGTCCTCAAAGCTCT
ACTCAGCCAGCTCACTAAGATCGATGGAGGCATTGAGGTCAAGGAATCACAAAGTTACGG
GGAAGGGAGCTCCGAGAGCCTCTTGACCGACATTGCCATTCCAAGCACTGCTTGGAATTG
ATGACGATGGTCGAGTGTAGTAGTGTGAGAGTCATTATTGCTATATTCATATTCATGATTGG
AACTATTCTTC

9) bra-miR172c-3p
(TCONS_00050888: gi|61608335|gb|AAX47049.1| AP2-like transcriptional factor)

TAATTCGAAGCAAAAGGGTCAAGATATGCGGCTCAAGATGAACCAACAAGATTCTCTTCAT
CCTAATGAGATTCTTGGATTGGGTCAAACCGGAATGGTTAACCATATCCCAAATTCAAATCT
CCAATTTCGGGGCAGCAGCAACATTGGTGGCGGAGGAGGATTCTCACTATTTCCGGTGGC
TGAGAACCACCGGTTTGATGGTCGGACCACGACGAACCAAGTGTTGGCAAATGCTGCAGC
ATCATCAGGATTCTCTCCTCATC

10) bra-miR395-3p (c70764_g5_i1: gi|4033353|emb|CAA11416.1| ATP sulfurylase)

GTGCTAATTGACACCTCATCAGCGAGTAGAAACGTTATTTCAGACCAATGTCCATTCACTTA
TCCACTAATTATTATTTTAACATTTATTTGATGTATTAATAAACGATCCACTAACTGTCTCTTA
ATTACAAGATTTTTGATTAATCAATGTGCCTGTAAGAAACGCAACAACACGACAAACTGCAT
CAATGGCTTCTTCAGCCGCCATCGTCCTCTCCGGTTCTCCTTTCCGATCATCACAAATCGA
TAGCCACCGTGTCTCCCGTCATGCATCTAGATCAGCCTTCGTCGTCTCTCT
CCGTCGACGAGGAGGTTCTCTCCGCCGCGGTCTCGCCGTTAAAGCGGCTCTGATCGAGC
CGGACGGAGGGAAGCTCATGGACCTCGTGGTCGAGGAATCGAAGCGGCGCGTGATGAAA
CGCGAGGCGGAGACGGTTCCCGTAAGGATCATGCTGAGTCGCGTGGATCTCGAGTGGGT
GCACGTGCTCAGCGAAGGCTGGGCGAGCCCGCTAAAGGGCTTCATGAGACAGTCAGAGT
TCCTCCAAACACTTCATTTCAACTCGATCCGGCTCGAAGACGGCTCCGTCGTCAACATGTC
GGTTCCGATCGTTCTCGCGATCGACGACGACCAGAAGAGTCGCATCGGCGATTCCGACCG
AGTCACGCTCGTTGACTCGTCTGGTAACCCTGTCGCCATCCTCAGCGACATCGAGATTTAC
AAGCACCCTAAAGAAGAACGTATAGCAAGAACCTGGGGAACAACGGCTCCAGGTCTTCCT
TACGCAGAAGAAGCAATCACCAGATCAGGAAACTGGTTGATCGGGGGTGATTTACAAGTC
CTGGAGCCGATCAAGTACAACGATGGACTTGACCGGTTCCGTCTGTCTCCGTCTCAGCTC
CGAGAGGAGTTCACAAAGCGTGACGCGGACGCTGTTTTCGCGTTCCAGCTTAGGAACCCG
GTACACAATGGTCACGCCCTTCTCATGACCGATACTCGAAGAAGACTTCTCGAGATGGGTT
ACAAAAACCCTGTCTTGTTGCTGAATCCACTTGGTGGATTCACTAAAGCCGATGATGTACCT
CTCAGCTGGCGTATGAGGCAGCACGAGAAGGTGCTTGAGGACGGTGTTCTTGATCCAGAG
ACAACAGTGGTTTCGATATTCCCGTCTCCTATGCATTACGCTGGTCCAACAGAAGTGCAGT
GGCACGCAAAGGCTAGAATCAACGCCGGTGCTAACTTTTACATTGTGGGTCGTGATCCAG
CCGGGATGGGTCACCCAGTTGAAAAACGTGATCTTTACGATGCTGATCATGGAAAGAAAGT
ACTAAGCATGGC

11) bra-miR158-5p
(Brara.C04365.1: Arabidopsis protein of unknown function (DUF241)

ATGGCTGCAACCTACCACGTTCGGTCTTGTAGCTTACCCGCTCGTCTACCCTCAAATGGTC
TCAACCATATTCAGCAACTTCTCAGTAAGCTTCCTACAGATAACAACAATAGCCTATCACTT
CTCTCACAGCTCTATGAATCCATCTCTCATCTCTTCAACGACTCACCAGCTTCATCACTACT
CCCTCATCACTCTTTCTTCACCCATCTTCTTGATCTTTCCCTTGTACACCTTGACTTGTGCTC
CAAGCTACGAGACATAACTTGCCGCATCAAGGATTGTCTCCGAGACCTTCGGTCTGCTTTC
AGACGGAGGGGACACGGTGGAGATTTTACGATCCGATGTCACGTTAAAGCCTTTGTACGC
TCTCGACGACTGATTCACAAGGATCTTGCTAAGCTCCTCTTATTGCTCAAACAAACAGATCA
TCCTTCCATCGAGTCAACTCATCCCTTGATCACACTTCTCCGACAAGTATGTTCCCAAACAT
GTCGTTCCTTTAGGACAGTCATGTTGTCCTTATCCTCATCAGTACCAAAACCCAGGCCTTCC
AGATGGGCTCTAGTTTCTAAACTGGTGATCAAGAACGTTACTAATACAAGTGCTCAAGTTCA
TAGCGGAGACAGAACCGAGTTCCAGATGATGGACGAAGAGCTGCAAAGATTCTGTTCAGC
GAAAGAGATAAAGAAGGAAGGAATCAAGTCTTTGATCGCCCTCTTGGATAACGTAGATGTT
GTAGTTGAAGATCTAGAGGAATCGCTTGAAAGCGTGTACAGGCGTATGATCCAAGCCAGA
GTCTCTCTTTTGAACATACTCTCTTTGCATATATAG

12) bra-miR161-3p
(TCONS_00015119: gi|297797589|ref|XP_002866679.1| Pentatricopeptide repeat-containing protein)

CCCAGTTGTCTCAAAGATACAGGATTTCTGGGATGAAAGCCTGGATCATGGTTTTGACCCT
TATTGTATAATCTGTAGCACTATGACAGGAAAACGAAGCTACTAGCAATAAGCTCAAGTCGA
CTCGCATCCCAAAAAAAAGAACCTGAAATTCCATAGGTCTGCACTAGCCTCTAACAATAACC
CCTTACGTGCTATCAGGAGGTCCTTCCGTTAGCAGTTCATATGTATGAGAGCTAAACTTGC
AGCCACTTTCCTCCATAACGGCGAACAGTTCACTAAACGCCTCCACCAGTCCCTGCTTCCC
AACACNNNNNNNNNNNNNNNNNNNNNNNNNNNNNNNNNNTAACGATCTTCCAAGCTATCT
CATCATGATAATAACCACACCGAAGCAAATTCTTGAAAACCCAAGCCCCTCTCTCGCTTTCT
CCGCTTTTATACAGCCCACATATCAAGATTTTGCAAGACTCTAGTTGCGGTATGTGACCAAC
ACAGATCATATCATCTACAACCTTTGCTGCTTCCTTAAACATTTCCAGCTTGCAGCAACAGC
TAAGAAAAGCACTGAAAGTGGACTCACTCGGTGATACTCCTTCCTCCTGTTGCATGCGCTC
AAGCACCTTTTGTGCAACTCTGAGATTCCCAGTTTCGCAGATCCCTTTGATCAGCATCTCAT
AGGATTTGGCGTTGGGAGTAACACCGTGTTCTACCATTTTGTCAAGAAGCTCAACAACAAT
GTCAAACTCAATCNNNNNNNNNNNNNNNNNNNNNTATTAGCCGTCAGACAGAGATCGCTCTC
TTTTCCATGTTTCATCTCTACCAGATGTTTGATCAGAGCCAGAAACGTATGCTGAGAAGGCT
CACAACCATCATCAAGCATACACTTTAGAACATCAAATGCAGAATCTGTCAGCCCTTGATCG
CCATAACCTTTAATCAGAGATGAATAAGTGATGGAATCTGGGAAAACACCATCCTCCTTCAT
CTTCCCCATCATATCCTCAGCTTCTTTCAGTTTCCCTGCGCTACAATAGCTTTGAATGAACG
TTGTGTAGGTATGCGCATCAGGCTTTGCTCCAGATAACAACATTTCCTGAAAACGTCTCTC
GGCATGATCAAAATCCCCTTCCTTCAGCATTCTATGAATCAAGATTGTCTCAGTAATGACTG
TAGTCTGCACACCCTTCTCAACCATTTTCTTCTCTAACAACATGGCTTCACTCAATTTTCCAT
CGGTGCATAAGCCATGAATCAGGGCGTTAAAAGTGGACGAGTTTGGCAAGCAACTCTTGC
TTAACATTTTCTCAAGGATAAGTTTAGCTTCTTCTAGTTTATCTGACTTGCAGTATCCATCAA
TCANNNNNNNNNNNNNNNNNNNNNGTGCAGTGTACATCACCACATTTGCATCTACTACACCT
TTCTCCTCGAGAGAATCAAACAGTTCGCGAGCTTCTTCTACTTTTTTCCTTTTACACAAAGA
ATCTATGAAACTGTTATAAGTCCANNNNNNNNNNNNNNNNNNNNNNNNNNNNNNNNNNNN
NNNNNNNNNNNNNNNNNNNNNNNNACCAAGCAACCTATATGCACTATCGAAATTACCAGA
CCTACACTGCGCATCAATTAATGAGTTGTAAGTGACAACACTAGGAGCCACTCTGCGCTCA
ACCATCTTATGAAACACTCCCATCGCTTTGTGCACATTCTTCCTACAGAACCCATGTATCAG
CTCATTGTAAGTGCGTGTATTCGGACTCACGTTACGCGATTCCATCAACTCCACAACGTCT
AGCGCGTCTTCCNNNNNNNNNNNNNNNNNNNNNNNNNNNNNNNNNNNNNNNNNNNNNNNN
NNNNNNNCTGCTCACAATACCCATTAATCAGTGCATTGTAAGTGACTACATTCGGCACCAA
CCCTTTCTCCACCATATCACCAAGCAACTCCCTCGCTTCCTCGAACTTACGTTCACTGCAA
GAACTACTAATAAGCACCGTGTAAGTGTGAATATTGGGTGTAATGCCTTTCTCCGACATCTC
CTTACGCAGGTCAAGAGCTTCTGACTTCCGTTTCGATCCACACAGAGCGTTTATAAGCACC
GTGTAAGTACGAACCGTTGGATAACAGTTATCCTCCTCCTTCATTTTCCCAAACAAGTCCAT
CGCTTCATCGACCCTCCTCGCTACACAAAGCCCGTGTATAAGATGAGTGTACGCAACCTCG
TTCCTTCTAAAACCTTTCGAAGCCATCTCCTCGAAAACCTTAAAAGCAGAGTCCAANNNNNN
NNNNNNNNNNNNNNNNNNNNNNNNNNNNNNNNNNNNNNNNNNAATACCCCATGATCAAA
GACGTATCCGTGAAGAAATCAGGTTCCAATCCAGCTTCAACGATCTTACTCACATACCGCT
TCGCCGTAACCACATTCCCCACCTTGCAATACCCAAAGACCATCTTGTTATAAGTGTAAACG
TTCGGAGAAACCTTATCATCCTCCATCATTTCCATATAAAGCTTCTCCATTTCATCAACCATT

**13)	bra-miR391-3p**
(TCONS_00063918: gi|297810021|ref|XP_002872894.1| Pentatricopeptide repeat-containing protein)

GCTTGCTGGTGGTGCTTCTGAAGGACCAGACCCGAGAGAAGTAGAGCTCTACGTGGAAGA
GATACTGTCTCTGATGCAGTTAGGTGAGGATTATACAGAGTTCATGGTTTCAAAGATCAAGT
CTTTGACGTCAGTTGATGCTGAGTTGTTGCCAAGGGCTACGAAGGCGTTTAGAAATGGTAG
CTTTAGCAAAGTGATTCAGGACGTGACGGGATTCNNNNNNNNNNNNNNNNNNNNNNNNNN
NNNNNNNNNNNNNNNNNNNNNNNNNNNNNNNNNNNNNNNNNNNNNNNNNNNNNNNNNN
NNNNNNNNNNNNNNNNNNNNNNNNNNNNNNNNNNCACTTCGATGGTGGACGATGTGTTC
TACGTGTTGCAGAGTTGTCTGAGGAGAGCGATTTCGACTTCGAACATCAGCTCTGTGATTG
CTGTGTTGAGCAATGCTGGTAGCTTGTTGGCTAATGATTACCATGAAGCTTTGCAGCAGAA
GATCAGAGAGCCTAACCTTGGTGCTAGGTTGTTCTTGGGTGGTATCGGTGTGGAGAACAC
TGGAACTGAGATTGCAACTGCTTTGAACAATATGGATGTGAGCTGCGAGTACATTCTTAAA
CTCAAACACGAAATCGAGGAGCAATGTACTGAGGTGTTTCCTGCACCAGCAGATAGAGAG
AGGATAAAGTCATGTCTATCCGAGCTAGGCGAGTTAAGCAACACGTTCAAGCAGNNNNNN
NNNNNNNNNNNTTACTCAACTCTGGCATGGAACAGCTAGTAGCAACCGTAACTCCAAGAAT
CCGTCCGGTTCTAGACACTGTAGCTACCATAAGCTACGAGCTGACAGAAACAGAGTACGC
AGAGAACGAGNNNNNNNNNNNNNNNNNNNNNNNNNNNNNNNNNNNNNNNNNNNNNNNN
NNACTTCTCCACTCGGTGGAAACAAACGCTGCGTGGCTCCAACCGCTAATGACTTCAAACA
ACTACGACTCGTTTCTGCATCTCATAATAGATTTCATAGTGAAGAGACTTGAAGTGATAATG
ATGCAGAAACGGTTTAGCCAGCTTGGTGGNNNNNNNNNNNNNNNNGCTTCAGCTAGACCG
AGACACGAGGGCTTTGGTTAGCCATTTCTCGGGTATGACTCAGAGAACAGTGAGAGATAAA
TTCGCTCGGTTAACTCAAATGGCGACGATACTGAACTTGGAGAAGGTCTCAGAGATTTTGG
ACTTCTGGGGAGAGAACTCAGGACCTATGACTTGGAGACTTACCCCTGCTGAGGTTAGAC
GGGTCTTGGGTCTTCGGGTCGAGTTTAAAGCCGAATCTATTGCTGCTCTCAAGTTGTGATG
TTACTTTCCTCTTTTATTTATGTTGTCAACCCACATATTACTCTGTTGCTGTATAACTTGTATT
GCCCATAGTGCATGATCAACGG

14) bra-miR396-3p
(Brara.I02082.1: PHOSPHATE TRANSPORTER PHO1 HOMOLOG 2-RELATED)

ATGAAATTCGGAAAAGAATTTTCTTCACAGATGGTGCCTGAGTGGCAGCAGGCTTACGTGG
ACTACAAGTATCTCAAAACCCTAATCAAAGATATCAACCGCTTCAAATGCAAAACCAATCCT
CAAGGCCATGAGATGAAAACGGCTCTGCACCGCACATTCATCGGTCAAAAGAGACATGGC
TTTGGCAGCGGTTGCGGTCAGATCAGCCCTTCTTCGACAGTGGTCGACATTAACGACGGA
ATAACGACGGCGCCAATTCATGTGAGTTCTTCGGTGACTCACCAGTACGAGACGACGTTTT
TTATGACGGCGGAGAGAGGAGGAGAGTATGAGTTGGTGTTTTTCCGGCGACTAGACGACC
AGTTCAACAAAGTGGAGAAGTTCTATAAAGAGAAAGCTGATGAAGTGGTGAAAGAAGCGGA
GGTGCTTAACAAACAGATGGATGCGCTGATCGCGTTTCGGTTTAAGATGAAGGAGGAGCG
TACGGCGGAGATGACTCTCATGGCCTCACCTAATGCGGTTTCTCCGGCGGAGCTCGCCAA
AACGTCTACTCAATCTCCCACGGGAGTCATAGAGGAAGGAGGCTCCAGTGGAGCTGGTCG
ATCCGATGAAAATGACAATAACGTTGAGAAAGACAGTTATAGCAAAACAACACTCAAACCG
GCCAATGACATGAGCAAGATGAAAGATACGACGAGACCAGCTTCGATGGGAGTTCTAAACT
CCGTCAGGATCAGCTACACTAAGGGAACGCCTAGTTCCACCATTAAAAGCGTTCTCAAAGT
CTCGAACGAGCCAGAGCTCACATTAAACAGATATAATTTAAGGAAAATCGAGGAGAAGCTA
AGATGTGCGTTCGTAGAGTTTCATCGGAAACTATGGTTTCTTAAAAGCTATAGCTTTTTGAA
TGTGTTGGCGCTTTCGAAGATATTGAAGAAGTATGATAAGGTAACTTCGAGGGATGCTGCG
AAGTCTTACATGACAATGGTTGATAACTCTTGCCTTGGAAGCTCTGATGAGGTTATGAGACT
CCTTGATCATGTTGAAACTACATTCATAAAGCATTTCACAAATGGCAACAGAACAAAAGGAA
TCAACATCTTGCAACCTAAAGCTAAAAGAGAGAGACACCGACTTACATTTTCCACAGGTTTC
TTGGGTGGATGCATGTTTTCCCTCGTTGTGGCTCTTGTCGCTATCGTTCGCACCCGAAACA
TCTTACAAGATGAACGCCAGGAACAGTACATGAACTCAATGTTTCCTCTTTATAGCTTGTTC
GGATTCATCGTGTTGCACATAATTATGTATGCTGCTAATATTTATTTTTGGAGGCGGTATGG
AGTAAACTATTCCTTCATATTTGGATTCAAACAAGGAAATGAACTCGGCTACAAACAAATAC
TATTCGTGGGATTCAGCATTGGTGCGCTTGCGCTTCTTTGTGTCCTTGCCAATCTTGACAT
GGAGACAAACCCCAAAACTAAAGATTACCAAGCATTAACTGAACTCCTTCCTCTTTGCCTCC
TCATTACTATGCTCATAGTTCTAATCCTACCATTCAACATTTTCTATCGATCAAGTCGCTACT
TCTTTCTCAATTGCCTATCTCGCATTCTTGCTGCTCCTCTTTGCAAGGTGACATTATCCGAT
TTCTTCTTGGCAGATCAATTATGTAGCCAAGCGCAAACTCTTCGAAGCATTCATTTCTACAT
ATGTTACTACGGTTGGGGAGACTTCAAACAAAGGCAAAACACTTGTAGAGAGTCACGAGTC
TTCAACACTTTCTTATTCATTGCTGCTGCCTTCCCATTCGTTTCTCGCTTCCTTCAGTGCATG
AGGCGAGTGTTTGAAGAGAGAAACATTGAACAAGGGTATAATAGTTTCAAGTACTTTTTAGT
AGTAGTTGCTGTTTGCTTAGGGATGGCTTATGAAGTTGATCGAAAGAAAGATAGTCAAACTA
TCTGGAGATGGTCAGGTGGGGTTACTTCTGCCATGGCTGTGGTTTTCTGTACGTACTGGGA
CTTGGTTCAAGACTGGGGACTTCTTAACCGAACATCCAAGAATCCGTGGCTACGTGATGAA
CTCCTAGTACCCCACAAAGAAGTTTACTTCATTGCCATGATCTTGAACGTGGTGCTGAGATT
TGCGTGGCTGCAAACAGTGCTAGATTTACAATTTGAATCAATTAACACACAGGCGGCGATT
GCCTTTGTTGCAAGTCTTGAGATCATTCGTCGTGGGATATGGAATTTCTTCAGGTTGGAGA
ACGAACATTTAAACAATGTCGGGAAGTTTAGAGCTTTCAAGACAGTTTCATTACCATTCAAC
TACGAGGAAGACCAAAAAATATTATGTGGCCTTAATTAG

15) bra-miR9555a-3p
(Brara.B01903.1: Leucine-rich repeat transmembrane protein kinase)

ATGCCTAGATTACGACGGCCTCCGTGTCTACTACTCACTGTCTGGTTCTTGTGTATTTACAG
TTTGGTTCACGTCGTTCGAGCTCAAAACCGAACCGGAACTATTACTCATCCGGACGACGCG
CGTGCGCTGAACTCGATTTTCGCGACTTGGAAGATCAATGCGTCGAATGATTGGAACATCA
GCGGCGAGCTTTGCTCCGGCGCCGCCACAAACGGCAATGTCGACGTCGATGACCCTGCC
TACAACCCTATAATCAAATGCGCGTGTACTTTCGCCAACTCCACCTGCCGCATCACCGCAC
TGAAGGTTTATTCAAAAGATGTTGTAGGAACTATACCTGATGAGCTATGGACTTTGGAACAC
CTCACCAATCTGAACTTGGGTCAAAACTTGCTGACAGGACCCTTGTCTCCTGCAATTGGGA
ACCTCACTCGAATGGAATGGATGACATTTGGGATCAATGCGTTGTCTGCTGAAATTGGGAG
TTGTACAAAGCTACAGCAAATCTACATAGATAGTTCTGGGCTTAGCGGGGAAATACCTTTAT
CATTTGCTAACCTCGTGGAGCTGCAACAAGCCTGGATGATGGATCTGGAAGTTACAGGTC
GGATACCAGACTTTATTGGAAACTGGACCAAACTTACTGTCTTGAGAATTGTTGGAACTGGT
TTGAGTGGTCCGATACCGTCGTCATTTTCCAACTTAACTTCTTTGACAGAACTGAGGCTAG
GAGATATATCCAATGGAAGCTCTTCTCTTGAATTCATCAAAGACATGAAAAATCTAAGTATAT
TAGTATTGAGGAACAGCAATCTCACGGGGGAAATACCATCTGATATTGGAGAATACTCAAG
CTTGAGCGTAGTTGATTTAAGCTTCAACAAATTACATGGACCAATACCGTCTTCACTTCTCA
ATTTAACTTCACTTATTCACTTGTTTCTAGGAAACAACACGTTGAATGGTTCCTTGCCCACTC
AAAAGAGTCAGTCTTTGAGCAATATAGATGTGTCGTACAATAATTTGTCTGGAAGTCTTCCT
TCGTGGGTCAGCTTACCAAACTCGAATTTCAACCTAGTTGCTAACAGCTTTACACTGGAAG
GTCTTGACAACAGGGTTTTATCAGGACTGAACTGTCTGCAGAAGAACTTCCGTTGCAGTGG
AGGCAAAGGAATCTATTACAACTTTTCAATCAACTGCGGAGGCCCGGATATAACGTCTGTT
AGTGGGGCACTATATGACAAAGATGACGCGGATCTTGGACCATCTTCGTTTTTCGTGAATG
CTGCTAGGAGATGGGCAGTTAGTAGCATTGGTCTCTTTGCCGGAAGTAGCAATAATAGATA
TATAGAAACTTTACTATCACAATTTACCAACACTTCAGACTCAGAGCTTTTTCAGACAGCAAA
ACTTTCTCCATCTTCCATAAGATATTATGGTCTGGAGCTCGAAAATGGAGTCTATAACGTCA
CACTTCAGTTTGCTGAAATACAAATGACAAGTTCTAACTCTTGGACAGGGCTTGGAAGACG
GAGATTTGACATTTATGTCCAGGGAAGACTTGTGGAAAAGGATTTTGATGTACGCAGAACA
GCTGGTGGCTTCACTGACAGGGCAGTTCGTAGAGAATATAAAGCAAATGTGACAGAAAATT
ACCTCGAAGTTCATCTTTTCTGGGCTGGAAAAGGAACATGTTGTATTCCTATTCAGGGTGCT
TATGGGCCTATAATATCGGCAGTCAGTGCAGCGGCAGATTTTACACCAACTGTGAGTAATA
AGCCACCATCAAAGAAAAATAATAGGACTGGTGTCATTGTCGGTGTCATTGTTGGCGTAGG
ACTTTTTAGCTTCCTTGCGGGCGTTGTTATCTTCACAATACGACAAAGAAGAAAGCCATACA
CTGATGATGAAGAACTGCTTAGTATGGAAATAAAGCCTTACACATTTACTTACTCAGAACTT
AAAAGTGCAACTCAAGATTTCAATCTCTCAAACAAGCTCGGAGAGGGTGGATTTGGGCCTG
TTTATAAAGGAAACCTCAAAGATGGAAGAGAGGTAGCTGTGAAGTTGTTGTCTGTTGGATC
TCGGCAAGGGAAAGGACAATTTGTTGCAGAAATTGTAACAATTTCTACAGTTCTACATCGAA
ACCTTGTGACTCTTTACGGCTGCTGCTTTGAAGGAGATCATCGTTTGCTCGTATATGAGTAT
CTCCCTAATGGAAGTCTCGATCAGGCACTATTTGGGGAAAAGAGTTTACATCTTGATTGGT
CAACACGTTTCGAGATATGCTTGGGAGTAGCCAGAGGACTAGTCTATCTCCACGAGGAGG
CGAGGGTTCGCATTGTACACAGGGATGTGAAGGCCAGCAACATTTTGCTAGACTCCGAAC
TGGTCCCAAAAGTTTCTGATTTTGGGCTGGCGAAACTATACCATGGCAAGAAAACTCACAT
AAGTACCGGAGTTGCAGGGACCATAGGCTATCTTGCGCCAGAGTATGCAATGCGTGGACA
TCTGACAGAGAAAACAGATGTGTATGCATTTGGTGTTGTGGCTCTTGAGCTAGTTAGTGGA
AGGCCAAACTCTGATGAGATCTTAGATGATGAGAAAAAATATCTTCTTGAATGGGCATGGA
ATCTACACGAGAAAGGTCGTGAAGTTGAACTGATAGATGATAGGCTAAGTGAATTCAATGT
GGAAGAAGTGAAACGAGTGATTGGCGTTGCTCTGCTATGCACACAAGCATCTCATTCCTTG
AGACCACCTATGTCACGAGTAGTGGCCCTGTTGTCAGGAGATGTTGAGGTCAGTGATGTC

ACTTCTAAGCCAGGCTACCTAACCGATTGGAGATTTGATGACATCACCAGAGATGTTGAGG
TCAGTCATGTCACTTTCGAGCCAGGCTATCTAACCGACTGGAGATTTGATGACATCACCAC
TTCCTCTCTCAGAAGCTTTCAAACCACAGAGACGAACACTTCTGGCTCCAAGATTTCACCC
CGAAAAGCCGACTCTGAGCCGATGCTTGGAGCCCAAGATCAGTTTTGGAAGAGAACAATG
TCTCTTCTCTGA

16) bra-miR164a/164e-5p
(TCONS_00044570: gi|15222818|ref|NP_175997.1| NAC domain-containing protein 21/22)
TTTCTCTCTCTTTCCCTCTTAACTAATTGTCCGCTAATCAAAAAGATCATCATCAAATATTATA
AACCTAAAATCTAACCATTCAGTGATCAAAGGATCTCAACAAAGAGAGAGAGAGAGGAGCA
TGGAGTCGGAGGAGGAGAAGGAGAGTAGTATAAGCATGGTGGAGGCAAAGTTGCCCCCA
GGATTCAGATTTCACCCAAAGGACGACGAGCTTGTCTGCGATTACTTGATGGCANNNNNNN
NNNNNNNNNNNNNNNNNNNNNNNNNNNNNNNNNNNNNNNNNNNNNNNNNNNNNNNNNNNN
NNNNNNNNNNNNNNNNNNNNNNNNNNNNNNNNNNNNNNNNNNNNNNNNNNNNNNNNNNNN
NNNNNTGGCAAGGATTGGTATTTCTACAGCCAGAGAGATAGGAAATACGCGACTGGGCTG
AGAACAAACAGAGCAACGGCCACCGGATATTGGAAAGCCACAGGGAAAGACAGAGCCATT
CTAAGAAAGGGCAAGCTTGTTGGGATGAGGAAGACATTGGTGTTTTATCAAGGTCGAGCTC
CCCGTGGTCGTAAAACTGATTGGGTCATGCACGAGTTTCGTCTCCAGGGTTCCTTTGATCC
TCCTACTCTGAACTCTCCAGAGGTACAGGANNNNNNNNNNNNNNNNNNGGAAGACTGGGT
CCTGTGTAGAGTGTTCCACAAGAACACACAAGGAGACAACATGGGAAGCTGTTTTGACGA
GACAGTCTCCGCTTCTCTTCCTCCACTGATGGATTCTTACATCAACTTTGACCAAGAACNNN
NNNNNNNNNNNNNNNNCCTCTTCTTACCTCGGTCATGATCAGCACTTCTTCACCAATGAGCA
AGTGCCCTGCTTCTCCAATTTGTCACAGAACCAAACCATACACTCAAACCTAACCGACTCA
GTCTCTGAACTCGAGACTCCTTGCAAGAACCCTATCCCCTTGTTCACTGGTGGTTCATCCC
CCGCCATGCTCCCAGGGCTAGATTCGTTCAGTTCATCAGATCAGATGGTCCTCAAAGCTCT
ACTCAGCCAGCTCACTAAGATCGATGGAGGCATTGAGGTCAAGGAATCACAAAGTTACGG
GGAAGGGAGCTCCGAGAGCCTCTTGACCGACATTGCCATTCCAAGCACTGCTTGGAATTG
ATGACGATGGTCGAGTGTAGTAGTGTGAGAGTCATTATTGCTATATTCATATTCATGATTGG
AACTATTCTTC

17) bra-miR395a-3p=bra-miR395b-3p=bra-miR395c-3p=bra-miR395d-3p (c70764_g5_i1: gi|4033353|emb|CAA11416.1| ATP sulfurylase)

GTGCTAATTGACACCTCATCAGCGAGTAGAAACGTTATTTCAGACCAATGTCCATTCACTTA
TCCACTAATTATTATTTTAACATTTATTTGATGTATTAATAAACGATCCACTAACTGTCTCTTA
ATTACAAGATTTTTGATTAATCAATGTGCCTGTAAGAAACGCAACAACACGACAAACTGCAT
CAATGGCTTCTTCAGCCGCCATCGTCCTCTCCGGTTCTCCTTTCCGATCATCACAAATCGA
TAGCCACCGTGTCTCCCGTCATGCATCTAGATCAGCCTTCGTCGTCTCTCT
CCGTCGACGAGGAGGTTCTCTCCGCCGCGGTCTCGCCGTTAAAGCGGCTCTGATCGAGC
CGGACGGAGGGAAGCTCATGGACCTCGTGGTCGAGGAATCGAAGCGGCGCGTGATGAAA
CGCGAGGCGGAGACGGTTCCCGTAAGGATCATGCTGAGTCGCGTGGATCTCGAGTGGGT
GCACGTGCTCAGCGAAGGCTGGGCGAGCCCGCTAAAGGGCTTCATGAGACAGTCAGAGT
TCCTCCAAACACTTCATTTCAACTCGATCCGGCTCGAAGACGGCTCCGTCGTCAACATGTC
GGTTCCGATCGTTCTCGCGATCGACGACGACCAGAAGAGTCGCATCGGCGATTCCGACCG
AGTCACGCTCGTTGACTCGTCTGGTAACCCTGTCGCCATCCTCAGCGACATCGAGATTTAC
AAGCACCCTAAAGAAGAACGTATAGCAAGAACCTGGGGAACAACGGCTCCAGGTCTTCCT
TACGCAGAAGAAGCAATCACCAGATCAGGAAACTGGTTGATCGGGGGTGATTTACAAGTC
CTGGAGCCGATCAAGTACAACGATGGACTTGACCGGTTCCGTCTGTCTCCGTCTCAGCTC
CGAGAGGAGTTCACAAAGCGTGACGCGGACGCTGTTTTCGCGTTCCAGCTTAGGAACCCG
GTACACAATGGTCACGCCCTTCTCATGACCGATACTCGAAGAAGACTTCTCGAGATGGGTT
ACAAAAACCCTGTCTTGTTGCTGAATCCACTTGGTGGATTCACTAAAGCCGATGATGTACCT
CTCAGCTGGCGTATGAGGCAGCACGAGAAGGTGCTTGAGGACGGTGTTCTTGATCCAGAG
ACAACAGTGGTTTCGATATTCCCGTCTCCTATGCATTACGCTGGTCCAACAGAAGTGCAGT
GGCACGCAAAGGCTAGAATCAACGCCGGTGCTAACTTTTACATTGTGGGTCGTGATCCAG
CCGGGATGGGTCACCCAGTTGAAAAACGTGATCTTTACGATGCTGATCATGGAAAGAAAGT
ACTAAGCATGGC

18) bra-miR403-5p (Brara.I03870.1; Plant protein of unknown function (DUF827))

CACAATGATAGCGAATCACTAGTTCAAGAGCTCTTTTCTTTATAATGTCTCCTTCTGCGCAG
TAACTATGATTACTGTGATGAGCTTCTCTTTAAATTTTCTTTACCTTTTGAGTTTTGACAAAC
CATTTTGTTGTTCTTGAGGCTGAGAATGGCTGAGAGGAGGCTTCAAAAGAACCCTGCGGC
GGAGGCCATTCCCGGTACACCGAGAATCCGGGATGTGAGAATCGTATCTGGGTCGGAGGT
AACCGGATTATGTAGTCGTGGGTCTTCTCGGGTTCCATTCAAACTCAAAGACAATTACGAC
GGTGTGGGAGAATATGACCTAAATAAAATGGAAGAAAAAGCAGCAAAGCTTGAGAAGGATC
TAATCATGAAAGAGTTAGAAACTCTAGATTTTCTAGAAGCTCTTGGATCAACAAAAAAGATC
GTGAAAGACTTGAAGCTGGAGCTGCAGCAGCAACAACCCATGAGATGCATGGAGAGCCCT
GAACATCTTCGTTCAGAAATCAAAGAGATGAACCATGATGAACGTTGCCATCGTACTCCCA
TGAGGTCTCCAGATATGATCTTCATGGAACTGAAGCAAGCCAATATGAATCTTGGTAAAAC
CATGGATGATCTTTCGATGATACGATCCTACGTTGAATCTTTGAATATGAATACGGTGGAAC
ATAAAGATTTTCTTGGGGTGGCGTCTCTAGCTGAGGAGCTCAACAGTTTGAGGTTAAAACA
TGATGATGAAGAGAGATTCATTACCAAGAATGTATCCGTGAACCCGCAGTGCGAACAAGTT
AAGATGGTTGTTGAAACCAATGATAACAAGCAGAGCAGCACATGTCTTAAAACCGCAAGAA
TGAGATTGGTTGCTGCAAGAAAAAATGGAGGAAGCAGCTAGAGCAGCGGAAGCACTTGCGA
TTGCTGAAATGACCATGCTATCCAGTGCCAAAGACCAAGACGAGTTTTGCTTCCCAGAGCC
ACCAAGGTCACCTTTAACACTCAAAGAACAAATGCAAGAAGACATATCCATTGAGATCATGA
GGAAGCTGGAGGAAGCTAATGAAGAAGTTAAACAAAGCCAACAAGCCTTAGAAACTGCGTT
AAACCGGGTGGAAATTGCTAACGTGAAGCAACTTGAAGCAGAAGATGCGGTTCGCCAGTG
GAACATTGAGTCATGGAAAGATCAGAAAGCTATTAAAGCAAAGCGGTCCATGAAAGATGAT
AACATTCCTCGACGCTCCTTCTTGAGCCATGTGAGCCAGCACGAGCCGCTTGACAATCTCG
CTAAGCCGATGCTAAAACGTAATGTTTCAGTCGGCAGTGGCCTAAACCGGAAACAAGTCCC
TAACACTGATGAGAAACATTTGAGGAGAAAGTTTAGATTCATGCACAACACGGTTCCTGAA
CAGACAGAGTAAGCAAAACCACAGCCTTTAATGAATGAAAACATGTTTTGCTCATTTCATTT
CGTTTTTTTTTAATTATCTTTTGTTTCTAATTCGTTTGATCATATATGAGTGTTGTTTGCTTCT
ATTATAAACAAACCATAATGATTATTGTGAAATGTGTCGTCTTGATGCAA

19) bra-miR5725 (c68837_g4_i12; gi|297793391|ref|XP_002864580.1| Kinase family protein)

ATCCTTACAGATACCGAGCTGCTGTCCTGATGGATGATCAGAGAGAGACAGAAGCAGTGG
AAGAGCTGTCAAAGGCTATAGCTTTCAGACCGGAACTGCAAACGCTGCATCTCAGAGCAG
CGTTCCACGAGGCTACAGGGAAGCTTTCATTGGCTGCGCAAGACTGCGAAGCTGCTCTCT
GTTTGGACCCTAACCACACGGAGACGCTTCATCTCTACAGCAGATCCAAGGATCAAGCTTC
ATCCATTGACAACACCATTGCTGGCTTAGATTAGTTCTAAGTGACATTTGGGTATCTAGAGG
GCACACCTAAACGGCAGGCTCCTTTCTCTTTAGTTTCCATCTGTGGTTATGATCTCCATCTC
ACTCCACCACCGTGGAGATAGCCTTGGCAGTGCTGCATCGGCTTCTATTCCAATGATCTCT
CTCAACTTCACCACAACCTTTCTCATCGTTGGCCGTTGAGCTGAGTATGGATTCATACACTG
CCTAACCACTCCCAACAACCTCTCAGCAACATCCTCCTGAAACGTTTTCAGCGTCGGGTCT
ACAAGATCTCTCGCGGGCTTGGGTTCAAGAAACAAAGAATCAGGGTCTGGGAGCCTCCCG
GTGATGATTTCAAACACAAGGGCGCCAAAGTTTAAGACGTTGGTTTGTGGGTCAAGCGTTG
AGTGTTCTAAAAGGTTCTTGCTCGAGGAGGATTCCTTGGAGGGTGAGATGAAGTTGAGGA
CTGAAAAGTCTGAGACTTTGGCGGCGTTGTCTTCGGTTAAGTAGATACAAGAGGAGTCCAG
ATTGGTGTGCAAGATGGGTGGGTTGAGGTTGTGCATGTGCTCTAGACAGTAAGCTATTCCC
ATGAAGATTCTGAGTCTTGTAGGCCAGTCCAAGTGCTCCGTATGTTGAGCTTTCATGAAAA
AGATCAGTTCAACAGTTTATAGATCAGATTGTGTTAAGTTAATAAAAAGAGTTTCTTGATGAT
GATGGAGTACTTACAGTGAAGATGCTCGGAGAGGGATCCATTAGGAGCGTATTCGAAAAC
AAGCATTCTGTGGAAAGGCTCGTTCTCATGGCAATACCCAATCACATTCAGAAAATTCTTGT
GGTCTACTTTGGCTAACTTACGTATCTTTTGTAGCAGCTGTGTTTCCATATCGGTAGACCAG
TTTGCACGAGAGCCAGCCGCGATGGAGACAACAGCAATTTCAGCACCAGTGGATAAAGTC
CCTTTATAGATGGTACCGTCTGAGAAAGAGCCAATGATGTTACTGAAATCTTCACATGCTGC
TTGCAGCTCTGGTAGCTGCATCCTCGGTACATCTGTGATAAGGGGATCTTGAAGCTGGCCA
CTGGTGGTCCACGGCTTGATAGGAATTTTTCTGGTGTGGATAAGAAAGAAGATGATTAATG
CCACTGAGACTGTGAACACGCCAACGACTATGCCCACGATCATAGAAATATTATGAGTCTT
TTTAGTTCTTGGAGGTGGAGACAAAAGTTGTGGAGGAGGTTCAGAAGGTGGGTTTGGGGT
TTGTGCTGTCCTGACACTACTAGGAGGAGGGCTTGGGGTTCCATCTCCTCCTGGTGGTGT
TTGAGTTTCTGGTGCTGGAGGTGGTTTTGTGGAGCCTTCAAATGCTTGATCAGGAGATGGA
GGGGAGGCACCCACAGCAGGACCTTTGCCCGGAAGAGGTGGTGTCTCTACCAAGCTGCG
ACGGTTCCAAGAAGCTTCGGATTGGATCAGTGAGTGATCTTGAGAAGGATCAAAGAC

20) bra-miR172d-5p (Brara.D00057.1; Beta glucosidase 10)

GAGACGGAAGAAACTAAAACAAAAAAAATGAAAGCTTTCTCTGTGTTATCCATTTCTGTGGT
CATCGTTTTGGCAACAAGTCACATTGATGCTTTCACAAGAGACGATTTTCCAGAGGATTTCC
TCTTCGGAGCTGCCACGTCTGCTTATCAGTGGGAAGGAGCTGTTGATGAGGATGGAAGAA
CTCCTAGCGTTTGGGATACTTTCTCACCCTTTGATAACATGGACAATGGAGATATAGCATGT
GATGGATATCACAAATACAAGGAAGATGTTAAGATAATGGCAGAAATGGGATTAGAAGCAT
TCAGATTATCTATCTCATGGTCAAGGCTTATACCTAATGGAAGAGGACACATCAACCCAAAA
GGTCTACTGTTTTACAAGAACCTCATCAAAGAACTACTAACCCATGGAATAAAACCACACGT
TACACTATACCACTATGATCTCCCTCAGGCTCTTGAAGATGAGTACGGAGGATGGATCAAC
CGCAAAATCATAGAGGACTTCACTGCTTTTGCAGATGTTTGCTTCAGAGAGTTTGGTGAAG
ATGTGAAGCTATGGACTACAATTAACGAAGCTAACATATTCGCCATTGGAGCTTACAGCGA
AGGAATATTGCCGCCAGGACATTGTTCTAATTCCAAATACGTCAATTGCTCCACTGGAAACT
CTTCAACTGAACCATATATTGCAGGCCATAACATATTGCTAGCTCATGCCTCTGCTTCAAAC
TTGTATAGACTCAAGTACAAGAGTAAGCAGAGAGGATCCATAGGCCTTTGCATATATACATA
TGGGTTATTTCCTTATACTAGCTCCAAGGAAGATGAGATAGCAACTCAGAGAGCTAAAGATT
TCTACTTTGGCTGGTTGTTAAAGCCTTTGGTGTTTGGTGACTATCCTGATGTGATGAAGAGA
GTTGTGGGATCTAGGTTACCTGTTTTCTCAGAGGAAGAGTCAGAGCAAGTTAGAGGATCAT
CTGACTTTGTAGGAGTTATCCATTACACGACACTGTATGTCACAAAGAGTAGACCCACGCC
TTCTATCCTTCCTAGCAACCAAAGTTTTTCCACAGACATGGGTGTAGAGACTATCTCCATTG
GGAACTCTGTTCCATGGGGTTTTGAAGGTGTCTTGGAGTATTTGAAACAGAGCTTCAACAA
TCCTCCTATCTACATTCTTGAAAATGGTTTAGCTACGAAACATGATTCGACGCTACAAGATA
CATCAAGAGTTGAATACATTCAAGGTTACATTGGTGCTATGTTGAACGCCATCAGGAATGG
ATCGGACACGAGAGGTTACTTTTATTGGTCGATGATAGACTTGTACGAGCTATTGGCTGGA
TACAGGCTCAGCTTTGGATTGTACTATGTGAATTTCAGTGATGCTGGTCTCAAGAGGTCTC
CAAAACTCTCTGCTTCTTGGTACTCTGGTTTTCTCAATGGTACAGTTGATGTTGCTCCTCGG
GATATTACTCAGCTGCAGAGCCACTCTTCTGGTTTGTCATCTTTGTGATGTTAGTTACCTAT
ATATCTAAAGATTCTGGTTAACAACACTCAAACACAACCACATAAGCTTCCCCCCGGAAGG
AAAATAATGTTTACATATTGTTCAAATAAGCAAAAGCGCATCTGGTGTAGTGGTATCATAGT
ACCCTCCCACGGTA

21) bra-miR160a-5p (c69810_g1_i2; gi|379323230|gb|AFD01314.1| Auxin response factor 17-2)

ATCTGTTCACGAGATGTTTATCGGTGTGAGGAGAGCTCCGAGCTCGAACAAAGGCGGCGG
GGGAGGAAGCTACTACGGCGATGAGTACTACGCCGGTTGCTACGGAGGAGGAGGAATTA
AGAAGGAAGACGGTGGCGAGAAGAAGGGGTTTAGTAGAGTTGGGATGGGGAAGCTGACG
GCGGAAGCGGTTTCGGAGGCGATTGGGAAAGCCTCGCAGGGTCTTCCGTTCGAGGTTGT
GTATTACCCCACGGCGGGCTGGTCTGACTTCGTGGTGAGAGCTGAAGACGTGGAGGCCT
CCGTGAGTGTTTACTGGGCTCCTGGAACTAGAGTCAAGATGGCGATGGAGACGGAAGATT
CCTCGAGGATCACGTGGTTTCAGGGCGTCGTCTCAGCTTCGTTTCAGGAGACGTGGAAAC
AGCTTCAGATCACATGGGATGAACCTGAGATTCTGCAGAATTTGAAGAGGGTGAATCCTTG
GCAAGTGGAGGTGGTTGCGAGCTCAACTCAGCTTCACGCCACTTACACTCCTCCAACAAA
GAGGTCCAAGTATCCACATGGCTCCTCAAGTGGGTTCTTGAGTGGAGGAGAGGAAGGAGA
GATGATCTATCCTGGAAGAGGACAAGCTATGGATCCAAATCACTATGGGTACACTACTTAT
ACTACAGTTCCTGCTGGCATGCAGGGAGCCAGGCATTACGAATTTGGGTCTTACAACAACT
CAACCGGATTCATTGGAGAAAACGCTCATCCTGAGTTCAACTTCTTTAGTCCTCTTCCCGGT
TTGGGAAGAGTCTCGACGACTCAGATGATGAACTTTGCCAGTCCGCCGTCAGATGACTTGT
CGCCTAATAGCAATACCACTAATGTTTCATCTGGAAATGACGCAGCTGGGAACAACAGAGG
CATTAGTTTTCAGCTGTTTGGGAAGGTGATAAACGTGCAGGAGCCTGCTGAGAGCGGTGTT
GCTGAGTCTAGCTTGTGCGAGGAGGATGGAAGCAAAGAGTCGAGTGACAATGAAGTTCCG
AATGAGGCCCAGTTGCAGGGTCATCATGAAGGAAGGTGCGGAGTCTAGGGTGCCTATAAG
ATTCCAGCAGACACAGAGGTAATAGCTGCAAAGGTGAAGGGAATGTTGGAATCTGTACGTC
CCAAGTAGTAGTGGTTGGTGGTAATAAAAAAAAAAGTATCTTATGGTTTTTCTTTTCTTTGCA
TTTTGACTTTCTCT

22) bra-miR161-3p
(TCONS_00015119: gi|297797589|ref|XP_002866679.1| Pentatricopeptide repeat-containing protein)

CCCAGTTGTCTCAAAGATACAGGATTTCTGGGATGAAAGCCTGGATCATGGTTTTGACCCT
TATTGTATAATCTGTAGCACTATGACAGGAAAACGAAGCTACTAGCAATAAGCTCAAGTCGA
CTCGCATCCCAAAAAAAAGAACCTGAAATTCCATAGGTCTGCACTAGCCTCTAACAATAACC
CCTTACGTGCTATCAGGAGGTCCTTCCGTTAGCAGTTCATATGTATGAGAGCTAAACTTGC
AGCCACTTTCCTCCATAACGGCGAACAGTTCACTAAACGCCTCCACCAGTCCCTGCTTCCC
AACACNNNNNNNNNNNNNNNNNNNNNNNNNNNNNNNNNNTAACGATCTTCCAAGCTATCT
CATCATGATAATAACCACACCGAAGCAAATTCTTGAAAACCCAAGCCCCTCTCTCGCTTTCT
CCGCTTTTATACAGCCCACATATCAAGATTTTGCAAGACTCTAGTTGCGGTATGTGACCAAC
ACAGATCATATCATCTACAACCTTTGCTGCTTCCTTAAACATTTCCAGCTTGCAGCAACAGC
TAAGAAAAGCACTGAAAGTGGACTCACTCGGTGATACTCCTTCCTCCTGTTGCATGCGCTC
AAGCACCTTTTGTGCAACTCTGAGATTCCCAGTTTCGCAGATCCCTTTGATCAGCATCTCAT
AGGATTTGGCGTTGGGAGTAACACCGTGTTCTACCATTTTGTCAAGAAGCTCAACAACAAT
GTCAAACTCAATCNNNNNNNNNNNNNNNNNNNNTATTAGCCGTCAGACAGAGATCGCTCTC
TTTTCCATGTTTCATCTCTACCAGATGTTTGATCAGAGCCAGAAACGTATGCTGAGAAGGCT
CACAACCATCATCAAGCATACACTTTAGAACATCAAATGCAGAATCTGTCAGCCCTTGATCG
CCATAACCTTTAATCAGAGATGAATAAGTGATGGAATCTGGGAAAACACCATCCTCCTTCAT
CTTCCCCATCATATCCTCAGCTTCTTTCAGTTTCCCTGCGCTACAATAGCTTTGAATGAACG
TTGTGTAGGTATGCGCATCAGGCTTTGCTCCAGATAACAACATTTCCTGAAAACGTCTCTC
GGCATGATCAAAATCCCCTTCCTTCAGCATTCTATGAATCAAGATTGTCTCAGTAATGACTG
TAGTCTGCACACCCTTCTCAACCATTTTCTTCTCTAACAACATGGCTTCACTCAATTTTCCAT
CGGTGCATAAGCCATGAATCAGGGCGTTAAAAGTGGACGAGTTTGGCAAGCAACTCTTGC
TTAACATTTTCTCAAGGATAAGTTTAGCTTCTTCTAGTTTATCTGACTTGCAGTATCCATCAA
TCANNNNNNNNNNNNNNNNNNNNNNGTGCAGTGTACATCACCACATTTGCATCTACTACACCT
TTCTCCTCGAGAGAATCAAACAGTTCGCGAGCTTCTTCTACTTTTTTCCTTTTACACAAAGA
ATCTATGAAACTGTTATAAGTCCANNNNNNNNNNNNNNNNNNNNNNNNNNNNNNNNNNN
NNNNNNNNNNNNNNNNNNNNNNNNNACCAAGCAACCTATATGCACTATCGAAATTACCAGA
CCTACACTGCGCATCAATTAATGAGTTGTAAGTGACAACACTAGGAGCCACTCTGCGCTCA
ACCATCTTATGAAACACTCCCATCGCTTTGTGCACATTCTTCCTACAGAACCCATGTATCAG
CTCATTGTAAGTGCGTGTATTCGGACTCACGTTACGCGATTCCATCAACTCCACAACGTCT
AGCGCGTCTTCCNNNNNNNNNNNNNNNNNNNNNNNNNNNNNNNNNNNNNNNNNNNNNNN
NNNNNNNCTGCTCACAATACCCATTAATCAGTGCATTGTAAGTGACTACATTCGGCACCAA
CCCTTTCTCCACCATATCACCAAGCAACTCCCTCGCTTCCTCGAACTTACGTTCACTGCAA
GAACTACTAATAAGCACCGTGTAAGTGTGAATATTGGGTGTAATGCCTTTCTCCGACATCTC
CTTACGCAGGTCAAGAGCTTCTGACTTCCGTTTCGATCCACACAGAGCGTTTATAAGCACC
GTGTAAGTACGAACCGTTGGATAACAGTTATCCTCCTCCTTCATTTTCCCAAACAAGTCCAT
CGCTTCATCGACCCTCCTCGCTACACAAAGCCCGTGTATAAGATGAGTGTACGCAACCTCG
TTCCTTCTAAAACCTTTCGAAGCCATCTCCTCGAAAACCTTAAAAGCAGAGTCCAANNNNNN
NNNNNNNNNNNNNNNNNNNNNNNNNNNNNNNNNNNNNNNNNNAATACCCCATGATCAAA
GACGTATCCGTGAAGAAATCAGGTTCCAATCCAGCTTCAACGATCTTACTCACATACCGCT
TCGCCGTAACCACATTCCCCACCTTGCAATACCCAAAGACCATCTTGTTATAAGTGTAAACG
TTCGGAGAAACCTTATCATCCTCCATCATTTCCATATAAAGCTTCTCCATTTCATCAACCATT

23) bra-miR5712 (TCONS_00068909; gi|80975776|gb|ABB54485.1| cDREB)

CTACACTCTTTCCCTACACGACGCTCTTCCGATCTCGGCGCTAATGATTTTCGCAGAGGTA
GGCTAACGCACGCCACCGCGCCAATTATATTAATATTATTATTTGCTCTCCCAGTCTTCATC
GGAGCTCTCCGGGTCGGGTAGTTTATTCAAGTCAACCCGTTCCAATAACCACCCGCCACTC
TTCAACCTCTTCTCATCTTCATCATCGTCTTCATGTTCTTCTCCATCACCGTTTTGATAATTT
CCACGAAAATTCTTGCTATCATCGCAACTCTCTTGGTCCTGACCAAAAACACGGTGGCGGT
TATCTAGCACCGCCGAGCCCAAAGCGTCTACCTGAGCACCCACCTCCGTTGCTTTCTTCCT
GATCGTCGCGGCCGACATATCCTCCTCGGTAAATTTCTCTCCGGTAAGAAGCTCAGGGAA
GTTGAGCCTCGCCGTCGGTCCTCTGAGGNNNNNNNNNNNNNNNNNNNNNNNNNNNNNNN
NNNNNNNNNNNNNNNNNNNNNNNNNNNNNNNNNNNNNNNNNNNNNNNNNNNNNNNNNN
NNACCTAGAGCGCTTGTTAGGCTCTCGAATCTCCGCAACCCATTTGCCCCACTTCCGCATC
CTTATACCCTTGTAAGGTCTCTCTCTCTTCCTCGTCGTCGTCGTCGTCCCGGAGACTGCCA
CGTTAGCTACTCCTCCGCTGCCCTCCATGGAGGATCTTGAAACCCTCCAAAGAAACAGACT
CTTAGAGGAATCGACCGACTTTTTCGATCTGGGTTTCGCCAGAGAGAGAGACTATGACACA
GAATAAATATGTCGGAGAGAGAGAGAGGAGGGGAAGGTTGCAGGTGGTGTTATGATGGTG
GATGGAGTAGTAGACAAATCCAAATTCACCGACAAGAGAAAAAGTAGAGAGAAGAGAATTT
TGGTTAGTTGGATTTTAAAGATAA

24) bra-miR9563a-5p (c60087_g2_i2; gi|297833846|ref|XP_002884805.1| Calmodulin-domain protein kinase CDPK isoform 2)

ATTAAACGCAAACGCGTTTTTCGAACTCATTAGATCATCTCCTTCAGCCTCTTCTTCTTCTTC
TTCTCCACGATTGACTTGTTAAACAAACTATACAAAAGATTACAAATCTTGAGGTTTTATGCA
TATGTAATTGCTATTAACCCATCCCTTCATTTTCCCTCCCTTATTTTCCGACCAAATAAAAAA
ATTGTTCTTTTCTTGTCCATCCCAGATCTTATTGATCTAAGATCATATTTCCGTTTTCTTGATT
TCGTCCTTCTGTGTTCATTTGAATTTTCGATTTTCACATCACCCTTCTGAAAAATTTCGGATT
TTTTTTATTCTTTGGCTCATTGATTATTCTTGTGGAAGACTTCTTTCTTTGCATTTAGTCTAGC
GACCATTGTCATTGGAGAAGAGAACCAAGTATCAGATTATTGAAGGATTTGACTTTTTCACT
GTCTCTGATCATTTAGTAAATGAGATCAGATCAGTGTGTTCTTTTGATCAGATCGGTGATGT
GAATGCGATTCTGGAGAACGTTGATTTAGGATTCATTATAAGTTAAGAGATGGGTAATGTTT
GTGTAGGACCAAACATTCCTGGAAATGGTTTATTGCAAACTGTTTCAGCTGCACTGTGGCG
GCCACGGATCGGAGCTGAGCAACAAGCTTCTTCCCATGGCAGCAATGCACAAGTTCCCAA
AGAAGCAGCTTCTTCCCATGGCAGCAATGCACAAGTTCCCAAAGAAGCAGCTTCTTCCCAT
GGCAGCAATGCACAAGTTCCCAAAGACGCAGCTGCTTCAGGACCAGAACCTGATCAAGTC
CAGAATAAACCCCCTGAGCAAGTCACTATGCCTAATCCTAGGTCCATTCCCGAAGCTGAAA
CCAAACCCAAACCCGAACCTGAAGAAGCCAAGAAGGAAGTTGGTGTGCAGGTGGAGACAA
CGAAGCCAGAAACAAAGCCTGAATCCATCCCGGAGTCAACAAAACCAGAAACAAAGAGTG
AAACCAATCCGGAGACGAAACCTGATCCCAACAAGCCTAAACATATGAGGAGATTGTCCAG
TGCAGGTCTCAGGACCGAGTCAGTGTTGCAGAGGAAGACCGAAAACTTCAAAGAGTTCTA
CTCACTGGGGAGGAAACTCGGACAAGGGCAGTTCGGGACTACCTTTCTATGCCTTGAAAA
AGGCACCGGGAAAGAGTATGCTTGCAAGTCCATTTCCAAGAGGAAGCTTCTGACGGATGA
GGACGTGGAAGATGTGAGAAGAGAGATTCAGATCATGCATCACTTGGCTGGTCATCCGAA
TGTTATATCCATCAAAGGAGCTTATGAAGATGTTGTGGCGGTACACCTTGTGATGGAGTTG
TGTTCAGGTGGCGAGCTTTTTGATAGGATTATCCAACGTGGGCATTACACTGAGAGGAAA

25) bra-miRX1 (braHC-mir-X33-5p; Brara.H03095.1; FZO-like)

GGGCCAAACGGATATTTTAGAGATAAGAAAACTAATCAGAAGCACAGACGAAATCTCTCTC
TCTCCATGAGAACTCTACTCTCTCACCGCCCATGTGTGACGTCACCTTTACTCATCTCCGCT
TCATTTCCGCCGTTTCCTCGCCGGTGCATTCGGTTATCATCATTTTCTCCTCCACGTCACAA
GCGTCTCTCTTCTCTCTCAATCAGAAACGCTTCGCTTGAATCCGCCGATCAAACTTCTCCCT
CTAGGCCGCGGACTCTTTATCCCGGTGGTTACAAGCGTCCCGAGCTCGCTGTTCCCGGTT
TACTTCTCCGCCTCGACGCGGATGAGGTTATGAGCGGGAACCGTGACGAGACTCTTGATT
TAATCGACCGCGCGTTAGCCAAATCGGTCCAAATCGTCGTGCTCGACGGCGGAGTTAACG
CCGGTAAGCTCTACGAGGCAGCTTGTTTGCTGAAGTCACTTGTGAAGGGCCGTGCTTACC
TCTTGATCGCCGAACGCGTTGATATCGCCGCCGCTGTTGGTGCGAGTGGTGTCGCTCTCT
CCGACGAAGGTCTTCCGGCGATAGTAGCGAGGAACACTTTGATGGGTTCAAATTCTGAGT
CGGTGGTTCTCCCTTTGGTGGCTAGGATTGTGAAGGATGTTGAGTCTGCTCTAACTGCCTC
TAGCTCTGAGGGTGCTGATTTCCTTATACTTGTTGGATCTCGTGAAGATCAACAATTGGCTG
ATTCTTTGTTGAAGAGCGTGAAGATACCCATTTTCGTGGTTTGCATAAGCAAAGGAGAAGC
TAAGGAAGAGTTGCAGTTACTGAGATCAGGAGCATCTGGTTTCGTTGTATCTTTGCATGAT
CTGCGCTCTTCTAGGGATGTTGCTCTTCGCCAGTTTCTTGATGGAGCTTCTTATGTTAACGA
GAACGAAACACCATTGGTTGAGGCTAGTGACCTGCATGAGAAGGCTGCTGGCTTGGTGAA
ATTTGAGGACAAGCAGAAAGAAATAATAGAAATGGAGAAATCAGTTTTGAGGGAGACCATT
GAAATTATCCACAAGGCGGCTCCACTGATGGAAGAAGTCTCCCTACTGGTCGATGCAGCTT
CCCGGATTGATGAGCCGTTTTTAATGGTTATAGTGGGGGAATTTAACTCCGGAAAATCAAC
TGTTATCAATGCACTTCTTGGGAAGAGATACCTCAAAGAGGGTGTAGTCCCCACCACCAAT
GAAATCACTTTTCTGTGCTACTCTGACTTAGAATCTGAGGAGCAACAACGTTGCCAAAGGC
ATCCAGACGGCCAATACATCTGCTATCTTCCTGCACCAATACTTAAGGATATCAATATCGTT
GACACACCTGGAACTAATGTGATCCTTCAAAGGCAACAACGTCTTACAGAAGAATTTGTTC
CACGTGCAGATCTGCTTGTTTTCGTTCTTTCTGCTGACCGTCCTTTAACTGAAAGTGAGGTT
GCGTTTCTCCGGTATACCCAGCAGTGGAAAAAGAAATTTGTGTTTATCCTGAATAAATCTGA
TATCTACCGTGATACTCGTGAGCTTGAGGAAGCTATCTCGTTTGTTAAAGAAAATACGCAAA
AGTTGCTTAACACAGAAAATGTGATATTGTATCCTGTGTCCGCACGGTCTGCTCTTGAGGC
GAAGCTTTCAGCAGCAGCTTTGGTAGGCAGAGATGATCTAGAGGTCTCTGATCCCGATTCT
AAGTGGAGAACCCAGAGCTTCAGTGAACTTGAGAAATTTCTGTACAGCTTCCTAGATAGCT
CAACTGTCACGGGGATGGAGAGAATAAGGCTTAAATTGGAGACGCCTATTGCAATTGCAGA
GCGTCTCCTTTCTTCTGTTGAATCTCTTGTTCTACAAGATTGCGTAGCTGCTAGGGAAGATT
TGGCTTCAGCAGACAAGATTATCAATCGTACTAAAGAATACACACTTACGATGGAATATGAG
AGCACATCTTGGAGAAGGCAGGCTCTGTCACTGATTGATAAGGCCAGATTGCAAGTTGTTG
ATCTAATAGAATCTACCCTGAGACTTTCTAGTCTTGATCTTGCAATCTCTTACGTGTTTAAAG
GGGAAAACTCAGCCTCTGTAGCGGCTACATCCAAAGTTAATGGAGAAATACTCGCTCCAGC
ACTCTCAAATGCTCAAGACCTACTTGGAAAATATGCTGAATGGCTACAATCAAATACTGCCC
GGGAAGGGAGTCTGTCCTTGAAATCATTTGAAAACAAATGGCCACAATATGTCAATTCAAAA
ACTCAACTGGGCATAGATACATACGACTTGCTTCGGAAAACTAATAAATTCAGCTTGAAAAC
CATCCAGAACTTGAGTGCCGGAACCACATCAAAACGGTTGGAACAAGATATTCGTGAAGTG
TTCTTTGTGACAGTTGGTGGGCTTGGAGCTGCAGGACTTTCTGCATCACTTCTAACCTCAG
TGCTACCCACCACATTGGAAGATCTTCTTGCTCTTGGCCTTTGCTCTGCTGGAGGGTATGT

GGCTATAGCAAACTTCCCATATCGCAGGCAAGCTATAATTGGTAAGGTGAATAAAGTGGCT
GATGCGTTGGCTCAACAACTCGAAGATGCTATGCAAAAGGATCTTTCGGATGCAACAAATA
ATCTAGTAAACTTTGTGAATATTGTTGCCAAGCCTTACCGAGAAGAAGCTCAGCTAAGACTT
GATCGTCTTTTAGGCATCCAGAAGGAACTATCAGATATTAGGAGTAAATTACAGTTGCTACA
AGTTGAAATTGATAACCTTCATGTATTAAGATGAGACTTTAGTTGCCCCAACAAAATGTCATT
TACATTGTTCACTGGGGACAAGTGTATGTGTTGTCATACATAATGAATAAGACGTACCAGTT
TCGACGAGTGAAATGGAGTCA

26) bra-miRX2 (braHC-mir-X75-3p; Brara.D02054.1; PHO2)

CAGGGCAACTCTCCTTTGGCTATCTCTTTCTTCGCCTCTTCTCTCTCAATGTCAAACCTAAC
AACCACTCCCTCACGAACCACCACCACCAGAGAGAGAGAGCAGCAGAGAAAAACAGAGAG
ACCCAGAAACAGAGGATAAAGCTGAAACAGAGAGAAACAGAGGATAGAGCAGAGAAACAG
AGAGAAGGAGATAAAGTTTTCGTTTTCCAATGGTTTGGAGGTTTGTTAATGGCGTAATAGAC
TCCTGGGACTGAAAGGGAGAAGCACTTACCCTTTGCTCATCTTCTTGATTTCTCACTTATTA
CCAAAAACCACTACGCTTCTTATGGTTAGATGGAAATATTTTAACTGATGGAGGAAACTCTG
GCTTGAGCAATTAACGAGCTTACCATCATGCTGGTCTCACTGCTACACTGGTGGGAGTATG
CGTGAATGAATGTCCAAGATACCGATCTTGTTTCTCACTTTGTTTCATTTTCTTGAACTACAT
CCTGTTTCCTCTATATATACTTAAAGATTCTAGCTCCTTTTGGGCAAATCTCCTTTGGCAAAT
AGTACAACTAGTGAAGGTTCTCCCCTTAGGTGACTTTGATACAAGTCATTTCTCTGTGGAGA
CTTTCACTTTGGGGCATATCTCCTTTGGCATTTTGGAACTCCAGTTACCTGTTGAGTTCCTC
CTTCTGAAGTTACTTCATTTCCATTTGTTTCGGCAAATCTCCTTTGGCATTATCAGAGTCTTT
CACTTGGCTCACCTTTATCTCTCTAGTCCCAGCAAATCTCCTTTGGCATTATCAGAGTCCAC
AGCTGACTCACGTTATCATCAGCAGAGCAAATCTCCTTTGGCATTATCCGAGTCTTACACTA
TCTATCAAAGTAACTTCAGGGAAGGAAGAGACACCATTTAATCTATTAAGGCATCTATAACT
ATGGATATGAACCTTACTGACTCCGATTGGGAGAGCTCCAGCGACAGTGGCAGCAGTGAA
CAAGAAGAAGTGGAGTTCTCTTACGGCGGACGTGCTCAAGACATCTTCTCAAACCTCGAAG
AGACCATTGGCAAAATCGATGACTTCTTGTCCTTCGAGAGGGGCTTCATGTACGGTGACAT
TGTGAGGTCCGCAGCTGAGCCCTCAGGACAGAGTGGCAGGGTTATCAACGTAGACATGTC
TGTCAATCTCGAGAGTATTCATGGGAAGGTTGTAAAAGAAGTTGACACCAAGAGGCTTCAG
AGGCTGCGTTCTATCTCACTCTGTGATTATGTGATCAACGGTCCTTGGCTAGGAAGAGTTG
ACAAGATAGTTGAGCGCGTCTCTGTCACCCTTGACGATGGTTCTAATTACGAGGTTCTTGT
AAGGAATCAAGATCAGCTTGTGGCTGTTCCTCCAAATATGTTGGAGGATTCTCAGTATACTT
ACTATCCAGGGCAGAGAGTTCAGGTTAAGCTGGCTCATGCTCCCAGATCAACTTCATGGCT
ATGTGGGAACTGGAGGGAGAATCAAGCTTTGGGAACTGTATGTTCTGTAGAAGCAGGACTT
GTCTACGTTGAGTGGGTTGCCTCCATCATCATGGGCGGTGGTGATAGGAACTTAACTGCA
CCTCAAGCTTTGCAGAGTCCTGAGAGTTTAACTTTGTTACCATCTGTTTCTCATGTAAGTTG
GCAGCTCGGTGACTGGTGCATCCTCCCTGGCGCTTCCCACTGTGATGCAGAAGAGCTGCA
AAAAGGTTTTAGCAGAAACATGCAGAGATCAAGCTCTGATGAGCTTTTTGTCATCACCAAGA
CGAAGATGAAGGTTGATGTTTTGTGGCAAGACGGTGGATGCAGCATGGGAGTTGATTCAC
AGCAGCTGCTTCCCGTTGGAGCTGTCAATGCTCATGATTTTTGGCCTGAGCAGTTTGTTGT
GGAAAAGGAAACTTGCAACAGCAAAAGATGGGGAGTTGTGAAGGTTGTTAATGCGAAGGA
ACAGACTGTGAAGGTGCAGTGGAGAACACTGGCTGAGAAAGAAGCAAGTGAGCAGATGGA
GGAAGTTGTCAGTGCATATGAACTTCTCGAGCACCCTGATTTTGGATTCTGTTACAGTGAT
GTGGTGTTCAGCGTTGCTACAGAGACGAAACACCAACTTACAGATAGTGACTACAGTGGTG
CTTATTGTTTGTCAAGCATTGGTGTTGTTGCAGGTTTCAGAAATGGTGTTGTGGAGGTGAAA
TGGGCCAATGGTTCTACTAGTGAGGTTGCACCATATGAAATTTGGAGGATGGAAAGGTCTG
AATTTTCCAACTCTAGCACTATAAGCTCTGCAGGTAGTGTTCAAGATCTAAGTCAGAAGATA
GCTCAATCAGATGAATCATCTTCAAACCATCAGGAAACGGGTCTAGTGAATCTCTACAGTG
TTGGTGAAAGCTGCAACAAGAACGTTCTGGAATCTAATAGTTCATTTTTCCTTCCAAAAGCT

GCCATTGGATTCATCACAAACCTAGCTTCAAGCCTATTCGGTTCTCATGGTTCTACCTCTGC
TATAAGTTCGCATTCACGCTGCAATGACACGGAAGATCAAAGCGACTCTGAGGTCCTTGTT
CAAGAAGCAACAGAACCACATGACATCTCTGAATCTAAATCAGATGAAGTGGATATGGACA
TGATGGTCAACTTGCCTATAGTAGAAAAAGGAATTAACAAGACACTAGATTCAACTATAGTG
AGCTTTAAACAGTTTGATATGGTTACTGACTGCTCAGACCATCATTTCTTTTCTCCGGGGAA
AGAGTTGGCACAATCTCCGGTTACAAAGAGTTGGGTGAAGAAAGTCCAACAAGAGTGGAG
CAATTTGGAGGCAGATCTTCCCAACACAATATACGTGCGTGTGTATGAAGAAAGGATGGAC
CTTATCCGTGCAGCCCTAGTTGGTGCCCCTGGAACACCATACCATGATGGACTTTTCTTTTT
TGACATAATGCTTCCACCACAGTATCCTCATGAGCCTCCAATGGTGCATTATCATTCAGGTG
GGATGCGACTGAATCCTAACCTGTACGAGTCAGGAAGAGTCTGCCTGAGTCTGTTGAATAC
ATGGAATGGCTCTGGCACTGAAATGTGGAACCCAGGGAGCTCCTCAATCCTTCAAGTCCTT
CTCTCGTTTCAGGCTCTTGTTCTGAATGAGAAGCCTTACTTCAACGAAGCTGGCTATGATAA
GCAGTTGGGCCGAGCTGAGGGAGAGAAAAACTCTGTGAGTTACAATGAGAATGCATTTCT
CATCACCTGCAAATCTATGATCTCGTTACTCCGCAAGCCTCCAAAGCATTTTGAGGTGCTT
GTGAAGGACCACTTCAAGCACCGAGCGCAGCATGTTCTGGCCGCGTGCAAGGCTTACATG
GAAGGCGTCCCTGTAGGATCATCAGCGAAACTTCAGGAGAGCTCAACCACAAACTCCACA
GGTTTCAAGATCATGCTCACTAAACTCTATCCAAAACTTGTCGAAGCATTCTCAGAAATTGG
AGTTGATTGCAGCCAAGGGGTTGCACTTGAACCATGAAGGTTTGACACTGTTAGTAACATC
TCCCTAACATCCAATAACTAATGTATAATATTGGTTTGCAAAGGATGGTAAGTCCCTTCCCA
TGCAGAATATGTTTTGTACTAAAAATGTGTGAAGAATAAAGAAGCCTGTAACATAGACTCAT
CTTGTATAATACCAAAAGGGTTGCCATGTACCATGTTTTGTAATAAAAATGAAATACTCGTG
TTT

27) bra-miRX3 (braHC-mir-X13-5p; Brara.G00856.1; Galactose oxidase/kelch repeat superfamily protein; Brassica rapa F-box only protein 6)

AGATAAAAAGAACAATACAAGACAACAGAAGAAGATGGTTTTAAGAAACAGTGAGAGGAGC
TTAAGCCCAAACCGTTTTTTTCTAAAGAATGGCTGCTACTACTGGTCCTGACTTGTGTGTGC
CATTGATTGTTTTCTCTCTCTCTCTCTCTCTTTTGTCTCTTTTGACGCGGGGCATGGAAGAA
GAGTTTGCCATGCTCAGACAGCTCATCGGACAGCTTCAAGAGCTCTTGCACAACAGCTCTC
CTCCTCCCCCTTCGCCTCCCTCCTCTTCGTCTTCTTCTCCGTCGTTTGTAGTTCTCCACAAC
CCTCAGCATCAGAACCGATGCTGCTGTTTGCCCTTATTTGACGACACTTCTTCTTCTGATGT
TTCTTGTGATACTCTAATGGCTGCTGGAAAGAGGCCCCGGTACTTGAAGATGTTAGATACT
GCCAAGCGTTCTCGAAAACAAAAGAATCAAGTAAAATCCTGCACGGAAGCAGGTGATGGC
ATCATGGATCAAGAACTCTGGCTGGAGTTTCCCCAAGATCTCTTTGAATCTGTTCTTTCCAG
ACTACCTGTCGCTACACTCTTCCAGTTCCGTTCAGTTTGCCGCAAATGGAACGCTCTCATC
GATTCAGATAGCTTCTCCAAATCCTGCACCGACCTTCCTCAGACCATCCCGTGGTTCTACA
CCATAACCCACGAGAATGTCAACTCGGGACAAGTCTACGACCCTTCTTCCAAGAAATGGCG
ACATCCTGTCATCCCTGCACTACCCAAGAAGAGCATCGTTTTGCCAATGGCTTCTGCGGGA
GGTTTAGTGTGCTTCCTCGACATTGGTCATCGCAACTTCTACGTGAGCAACCCTCTGACCA
AGTCCTTCAGAGAGTTGCCTGCTAGGTCGTTTAAGGTATGGTCTCGTGTTGCGGTAGGAAT
GACTCTTAACGGAAACTCCACCAGCGATGGGTATAAGGTCTTGTGGGTTGGGTGCGAAGG
AGAGTATGAAGTCTATGACTCCTTGAGAAACGTGTGGACCAAACGAGGGACCATCCCGTC
CAACGTAAAGCTCCCAGTACTGCTCAACTTTAAGTCACAGCCTGTGGCTATTAACTGCACG
CTTTACTTCATGTTAACGGATCCGGAAGGGATACTGTCCTACGACATGGTCTCTGGGAGAT
GGAGGCAGTACATCATACCGTGTCCGCCGCACCTGAGCGATCACACGCTAGCTGAGTGCG
GAGAGAGGCTGATGCTGGTGGGTCTTGTGAGCAAAAACGCAGCCACGTGCGTTTGCGTAT
GGGAGCTGCAGAAGATGACGCTGCTGTGGAAGGAGGTTGACAGAATGCCAAACGTATGGT
GCTTGGAGTTTTACGGAAAGCACGTTAGGATGAATTGTCTAGGCAACAAAGGTTGTCTGAT
GTTGTTGTCCTTGAGGTCCAGACAGATGAACCGTCTGATAATGTACGATGCTGTTGCTAAG
GAGTGGGGCAAGGTCCCTGGATGTACCGTTCCTCGTGGGAGGAAGAGACTTTGGATCGCT
TGCGGGACAGCGTTCAACCTTCCCCTACGGCCAGGGCTTGATCTTCTCTCTTCTCTCTTCT
CTCTTCTCTTCTCTCCTCTCTTCTCTTGTTCAGTTTTATGTACGGTTGGTTTGTTTGTTTTGA
TTAGTGGGACTGTAATTGTAATATAGAAAAACATTTGGTATTGTAATGAGTAACTTGGGCTG
TGTTATAACCGC

28) bra-miRX4 (braHC-mir-X15-3-3p; Brara.B00890.1; BREVIS RADIX-like 4)

ACCATAAAAATCTTTCTTTCGTTTTAGGTTTCTCAAAAGGTCAGAGCCGGTTTTGCATGTAT
CGATAATTGAATCCTTAAGCACACTTGAAATACTTGACTACAATGAACAGCCTTTGATACAT
CTGAAAGTCTAAATCTGCAAGCAGCAATAATGTAAAATCTCTCGATTTAAGCTACATTGACA
AGTGGAATCCCGCTCAAGTTGTCTTACTTAATCAGTAGACCAAACAAAATGGTTTACTTTTA
GCATTAACCGAGTGTGATTAGATTAGCGGATTTACCGTAGAGATGACGCGGGACCTTTTCT
TGATTTTTAATTAAAAAAATGGAACCGGTCTTCCTGGTTAAAGTGAGTATAAAAACATAAAAA
GAGCACCATGACCAAACACACAACACAAACCAAAGTTAGAAACTGACAGCATCTACTGTCT
CTCCGTATCCAAAAAGAGAAGAAACAAAATCACATTACCAAGAAACAGCAGCTGCCGGAAA
ATGCTGACTTGTATAGCTCGTTCGAAGCGAGCCGGCGATGAAACCTCGGGTCAACCCGAC
GATCCGGATTCCAAGCAAGCCAAATCTCTAACATCTCAGCTCAAAGATATGGCTCTGAAAG
CTTCCGGTGCTTACCGACATTGTACGCCGTGTACGGAGGGGGAAGTGCAACCGCCGATCA
AGAGCACTCCGGTGACGACAAAGTCTGATCCCGAATCTGATCAACGGTTTAAAATGCTGTA
CGGTAGATCAAACAGCTCGATAACAGCCACGGCGGCGGCGGCGGCGACGCAACAGCAAC
AGCCTAGAGTTTGGGGAAAAGAGATGGAGGCGAGGCTGAAAGGGATATCGAGCGGCGAG
GCGACTCCGAAGTCGGCGAGCGGGAGGAACCGGGTAGACCCGATAGTGTTCGTGGAGGA
GAAAGAGCCAAAGGAGTGGGTCGCTCAGGTGGAGCCAGGTGTTCTCATTACGTTCGTGTC
TCTTCTCGGCGGTGGTAATGATCTCAAGCGGATACGTTTCAGCCGAGACATGTTCAACAAG
TTACAAGCGCAAAGATGGTGGGCAGATAACTATGACAAAGTGATGGAACTTTATAATGTTC
AAAAACTAAGCCGCCAAGCCTTTCCCCTTCCCACGCCTCCTAGATCCCAAGACGAGAAGG
AGTATCATCCAGAGGATACTCCTACAACACCGCCTTTAAACAAAGAACGGTTGCCTCGCAA
CATCCATCGTCCAGCTGGATTGGCTGGTTGCTCATCTTCGGATTCCCTCGACCACAGTTCA
ACCCAAAGCCGGCAGTTTAACGACTCTGGAATACTAAACTCAACTCCTAAACTCTCAAGCC
TAAGCGGAGCCAAAACAGAAACATCTTCCATAACAAGCTGCTCGTCCAGAGACGCAGACC
GGTCAGAAGAGATGTCTGTAAGCAATGCGAGTGATGTTGATCAGAACGAGTGGGTGGAGC
AAGATGAGCCTGGCGTTTATATCACCATTAAAGTCTTACCATGTGGGAAAAGAGAGCTTAG
AAGAGTCAGATTCAGCCGAGAGATATTTGGGGAGATGCATGCGAGGGTATGGTGGGAAGA
AAACAGGGCAAGGATACATGAACAATACTTGTGATCATCTTCTGGTTTAGATGCATTAAACC
GGTTTTGTGTTTTTTATTTTTTTCTCATTCTCGGTATACTGTTTATTCCAAGCTGAAGTAAAAA
GAGAACCCTTCCGTAAGCACTCCTTGTGCCTCCTAGTCACACATATTTTAAATATCAACTGC
GGAATATGTTTAAGGAGGTTTCATATTTTTTTTCCTTAACAAAAGAAAATGCTTAAGCTTGGA
ATATATAGTGTTGGCTGGTTTGCTCAATATATGTATCGTTGGT

29) bra-miRX5 (braHC-mir-X18-3p; Brara.K00675.1; CBL10; calcineurin B-like protein 10)

ATGGACTTGCCGGGAGTTTCCTCTAGATCAAGCTCTCTTACCGTAGGAGAGCAACTCTGCG
TCGTCTTTATACCTCTCTTCGTCATTTTTGATTTCCTTTTCTCCACCGTTGGACAATGCTTCG
ATTGCCGCAGCCGCTGTTCGCCTCAGATATTTTCAGTCAACGAGGTTGAAGCATTGTATGA
GTTATTCAAGAAACTTAGTTGTTCAATTATTGACGACGGGTTGATTCACAAGGAAGAGCTTC
AGCTAGCGCTTTTCCAAGCTCCACATGGCGAGAACCTATTTCTTGATAGGGTTTTTTATTTG
TTCGATGAAAAGAAGAATGGGGTTATAGAGTTTGAAGAATTCATCCATGCCTTGAGTGTGTT
TCATCCCTACGCACCAATCGAGGAGAAGATCGACTGTAAGAATCATAGCACCATAACTATT
CAATGTCCTACTATTAATAATATACCGCTTTATGATCTAAGACAAACCGGATTTATTGAGCG
AGAAGAGGTGCATCAGATGGTGGCTGCTATTCTTATGGAATCTGAAATGATTATGTCTGAT
GAACTTCTGACCATGATTATTGATAAAACATTTGCTGATGCAGATTCTGATGAAGATGGTAA
AATAAGCAAAGAAGAATGGAAAGTGTATGTGCTTAAGCATCCCACCTTGTTGAAGAACATG
ACTCTTCCTTATCTTAAGGATGTGATGACAGTATTCCCAAGCTTTATATTCAACACTGAGGT
TGAAGACTAA
